# Ultrasound‐mediated therapies for the treatment of biofilms in chronic wounds: a review of present knowledge

**DOI:** 10.1111/1751-7915.13471

**Published:** 2019-08-07

**Authors:** Gareth LuTheryn, Peter Glynne‐Jones, Jeremy S Webb, Dario Carugo

**Affiliations:** ^1^ Faculty of Engineering and Physical Sciences University of Southampton Southampton UK; ^2^ National Biofilms Innovation Centre University of Southampton Southampton UK; ^3^ Institute for Life Sciences University of Southampton Southampton UK; ^4^ Centre for Biological Sciences University of Southampton Southampton UK

## Abstract

Bacterial biofilms are an ever‐growing concern for public health, featuring both inherited genetic resistance and a conferred innate tolerance to traditional antibiotic therapies. Consequently, there is a growing interest in novel methods of drug delivery, in order to increase the efficacy of antimicrobial agents. One such method is the use of acoustically activated microbubbles, which undergo volumetric oscillations and collapse upon exposure to an ultrasound field. This facilitates physical perturbation of the biofilm and provides the means to control drug delivery both temporally and spatially. In line with current literature in this area, this review offers a rounded argument for why ultrasound‐responsive agents could be an integral part of advancing wound care. To achieve this, we will outline the development and clinical significance of biofilms in the context of chronic infections. We will then discuss current practices used in combating biofilms in chronic wounds and then critically evaluate the use of acoustically activated gas microbubbles as an emerging treatment modality. Moreover, we will introduce the novel concept of microbubbles carrying biologically active gases that may facilitate biofilm dispersal.

## Graphical abstract

This review article discusses current practices used in combating biofilms in chronic wounds, and critically evaluates the use of acoustically activated gas microbubbles as an emerging treatment modality. Moreover, it introduces the novel concept of microbubbles carrying biologically‐active gases that may facilitate biofilm dispersal.

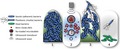

## The bacterial biofilm: development and aetiology

**Figure 1:**
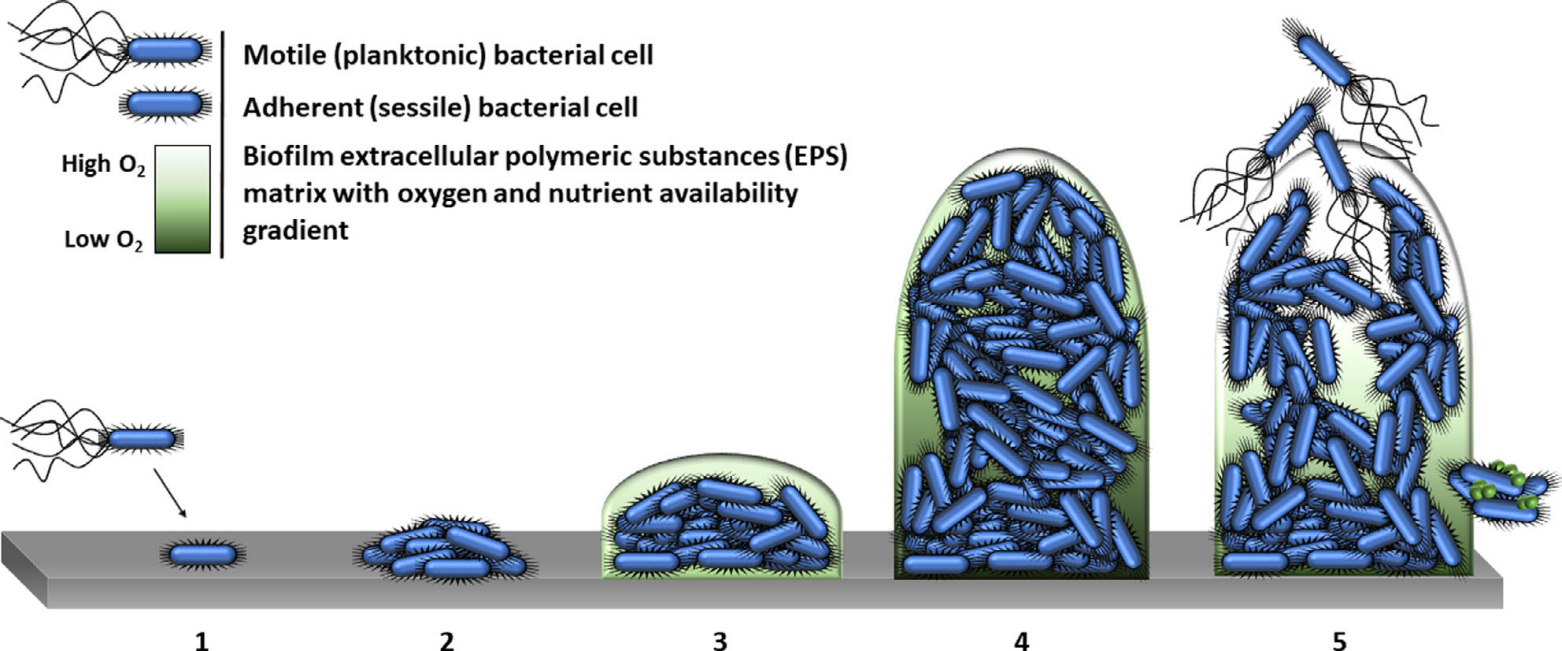
A simplified schematic representation of the sequential biofilm formation described for *P. aeruginosa*. (1) Initial attachment can be transient, but this association can become robust and lead to the aggregation of cells (2). This stabilized attachment leads to the production of extracellular polymeric substances (EPS), which encapsulate aggregated cells forming microcolonies (3). The maturation of the biofilm structure is achieved through intercellular signalling (4); a mature biofilm commonly features a concentration gradient of oxygen and nutrient availability. Oxygen is consumed by biofilm cells at a faster rate than it can diffuse in, which coincides with the gradient of nutrient availability. Consequently, the majority of metabolically active cells are located at the periphery of the biofilm, whilst persister, dormant or dead cells are found at the biofilm–substratum interface. In its final stages, the biofilm undergoes programmed dispersal of cells, which includes cellular mass and EPS sloughing off (5).

Though the microbial world is vastly diverse, the development of a biofilm remains perhaps the most ubiquitous means by which microbial cells can thrive within their given environment (Wu *et al*., 2015; Flemming *et al*., 2016). A biofilm can be described as a localized aggregation of microorganisms in a heterogeneous, sessile community, embedded in a dynamic matrix of extracellular polymeric substances (EPS) (Singh *et al*., 2017). From biogeochemical cycling in the ecosystem and the human microbiome to biofouling and disease, biofilms are simultaneously an essential part of life and a prominent concern for industry and public health (Donlan, 2002; Flemming *et al*., 2016; Kuliasha *et al*., 2017). The gross architecture of the biofilm is complex; proteomic investigation has shown that at least in *Pseudomonas aeruginosa*, biofilm development is regimented and sequential (Fig. 1) (Hall‐Stoodley *et al*., 2004). Though the specific stages of biofilm development have not been characterized for each prokaryotic organism individually, it should be noted that the development archetype is markedly conserved *in vitro* (Figueiredo *et al*., 2017; Lohse *et al*., 2017; Bartell *et al*., 2019).

The initial stages of biofilm formation are characterized by an impermanent association of planktonic cells with a surface, which is superseded by a robust adhesion and the production of EPS (Hall‐Stoodley *et al*., 2004; Holt *et al*., 2017). Although proteins, carbohydrates, lipids, DNA, RNA and water are stable constituents of the EPS (Taglialegna *et al*., 2016), there is a notable variability in the quantity of each macromolecule present between biofilms (Flemming *et al*., 2007). The mucoid phenotype of *P. aeruginosa* is found extensively in the cystic fibrosis lung and usually arises from the overproduction of the exopolysaccharide alginate (Schurr, 2013). This serves as a good example of the individual differences seen in the EPS of biofilms, exemplifying that variation can be both species‐specific and influenced by the immediate environment (Maleki *et al*., 2016). Adherent cells will then begin to propagate and assemble into microcolonies; the initiation of this process is attributed to monomeric adhesins (Jamal *et al*., 2015). Aggregation and microcolony formation in *P. aeruginosa* are attributed to the Type IV pili surface adhesin, whereas in *Staphylococcus aureus*, it is thought to be induced by polysaccharide intercellular adhesin (Persat *et al*., 2015; Maleki *et al*., 2016). Maturation of a biofilm into its distinctive three‐dimensional structure is achieved through sophisticated cell–cell communication termed quorum sensing (Laganenka and Sourjik, 2017). Quorum sensing is facilitated by the production of auto‐inducer signalling molecules, which allow microbial cells to determine cell density and collectively adjust gene expression in response (Rutherford and Bassler, 2012). This ensures the formation of an encapsulating extracellular matrix, with water‐filled channels for the transport and exchange of nutrients and waste products within the biofilm (Parsek and Singh, 2003). Programmed dispersal of microbial cells is the final stage of biofilm development, which is achieved by either the release of newly formed cells from biofilm aggregates or detachment of constituent peripheral cells by species‐specific saccharolytic enzymes (Marsh and Zaura, 2017). Erosion and sloughing may also occur due to mechanical (i.e. shear) stress upon the biofilm, which causes peripheral cells to disengage indiscriminately from the biofilm and enter the local environment (Rmaile *et al*., 2014; Jamal *et al*., 2018). Though dispersed cells regain motility, they remain physiologically unique from cells in the planktonic and biofilm phase; these dispersed cells are highly virulent in nature towards macrophages, which is a useful attribute given their main purpose is the colonization of new sites (Chua *et al*., 2014). Dispersion represents one of the most virulent stages in the biofilm life cycle, but perhaps also one of the easiest to target and thus potentiate killing of microbial cells (Hall and Mah, 2017).

## Significance of biofilms in chronic wound infections

The causality between pathogenic microorganisms and infection has been understood for over a century; yet, most research into the pathogenesis of microorganisms has focused solely on acute infection by planktonic cells. Over the last decade, this focus has shifted; greater emphasis is now placed on the role of multidrug‐resistant (MDR) organisms and biofilms, which mediate over 90% of chronic wound infections (Attinger and Wolcott, 2012; Bjarnsholt, 2013). The augmented persistence of biofilms can be attributed in part to inherited genetic antibiotic resistance traits, which actively reduce the efficacy of an administered antimicrobial agent. This commonly includes the use of membrane‐associated efflux pumps, which prevent antimicrobial agents reaching lethal intracellular concentrations, and antibiotic degradation enzymes such as beta‐lactamase, which alter the pharmacokinetic properties of beta‐lactam antibiotics (Høiby *et al*., 2011; Geisinger and Isberg, 2017). However, of particular concern is that when compared to their planktonic counterparts, it has been conclusively shown that the biofilm phenotype confers an innate physical tolerance to antimicrobial agents (Hengzhuang *et al*., 2012; Algburi *et al*., 2017). It is also noted that the extracellular polysaccharides within the dynamic EPS matrix potentiate this effect by acting as a shield, compromising the ability of the host immune system to detect the biofilm infection (Limoli *et al*., 2015; Kumar *et al*., 2017). Moreover, the production of extracellular toxins and lytic enzymes facilitates destruction of local immune cells, which provides a source of cellular components that can be utilized by microbial cells (Cooper *et al*., 2014).

It is evident that current treatment options available for biofilms are limited in both availability and effectiveness; thus, seeking to resolve a chronic infection by eradicating a competent biofilm formed in a wound bed is a multifactorial challenge. In response to a wound derived from acute trauma, tissue will undergo a sequential process of reparation that results in the reinstitution of anatomical integrity (Clark, 1993). Under normal physiological conditions, the progression of wound healing can be broadly categorized into four phases: haemostasis, inflammation, proliferation and remodelling (Guo and DiPietro, 2010). The phases within the process of wound repair are not mutually exclusive, but involve dynamic integration of cellular processes that overlap temporally (Robson, 2004). Diabetic foot ulcers (DFU) are a prevalent example of a chronic wound; commonly arising from comparatively minor trauma to the foot, they have been strongly associated with substantial morbidity and mortality (Walsh *et al*., 2016). The key pathophysiological difference between a common acute wound and a chronic wound is that the latter is typically associated with recalcitrant infection, ischaemia of the tissue and a prolonged or arrested inflammatory phase (Wolcott *et al*., 2008). One of the hallmarks of a chronic wound is high microbial burden and diversity, which is routinely attributed to the formation of poly‐microbial drug‐resistant biofilms in the wound bed (Banu *et al*., 2015). Given the pathogenicity and associated virulence factors of biofilms, there is credible evidence that they are implicit in preventing normal mechanisms of wound healing (Malone *et al*., 2017). It is reported that every 30 s worldwide, there is a lower‐limb amputation as a direct result of DFU (Yazdanpanah *et al*., 2015). This is undoubtedly accompanied by significant physical and emotional stress, as well as an increase in mortality rate (Costa *et al*., 2017). In addition, there is an undeniable economic burden associated with the cost of health care, from disease management to major intervention (Walsh *et al*., 2016).

## Current treatment strategies for biofilms in chronic wounds

The most established treatment for the removal of necrotic tissue and biofilms from chronic wounds is sharp debridement, but this mechanical method of biofilm disruption lacks both efficiency and effectiveness (Cooper *et al*., 2014; Yazdanpanah *et al*., 2015). Although the debridement of chronic wounds in clinical trials is largely concurrent with a reduction in the surface area of a wound, the period over which intervention is required is typically in the order of weeks to months and does not significantly correlate with complete wound closure (Williams *et al*., 2005; Rhoads *et al*., 2008; Wolcott *et al*., 2009; Yazdanpanah *et al*., 2015). Studies have shown that debridement can expedite wound healing by stimulating re‐epithelialization of the tissue; however, complete healing is typically observed in less than 20% of patients (Cardinal *et al*., 2009; Kim *et al*., 2018). The presence of persister cells allows the regeneration of the biofilm within the wound bed, which means that debridement is by no means a complete or permanent solution (Lebeaux *et al*., 2014). The efficacy of debridement can be improved by chemical and biological adjuvants, such as hydrogen peroxide and enzymes respectively (Watters *et al*., 2016). By causing the EPS matrix of the biofilm to degrade, and thus removing its principal means of protection and nutrition, the rate of wound healing is significantly increased (Kim *et al*., 2018). The physical perturbation of the biofilm caused by debriding has also been shown to temporarily restore antibiotic sensitivity; as the biofilm begins to regenerate, key antibiotic targets such as cell wall synthesis (glycopeptides) and protein synthesis (aminoglycosides) become viable (Wolcott *et al*., 2009; Hall and Mah, 2017).

Quorum sensing is an important regulator of biofilm development; it is the principal means by which microbial cells communicate within a given environment (Miller and Bassler, 2001; Rutherford and Bassler, 2012). The ability of microbial cells to carry out such sophisticated communication is a potent advantage; therefore, quorum sensing can also be considered as a valuable therapeutic target (Singh *et al*., 2017). By incapacitating this signalling mechanism, the regulation of gene expression, essential metabolic processes and virulence can be irreparably altered (Khmel, 2006). Natural and synthetic inhibitors of quorum sensing, such as furanones and Manuka honey, work by downregulating four major quorum sensing genes, which in turn has downstream consequences for genes associated with the biofilm phenotype (Wang *et al*., 2012; Jakobsen *et al*., 2018). However, it is important to note this is principally a method to render a biofilm more inert; it does not offer an immediate solution to detachment or physical eradication (LaSarre and Federle, 2013).

An additional means of controlling biofilms in chronic wounds is impeding their attachment to surfaces; one way in which this has been achieved is with the iron‐chelating glycoprotein, lactoferrin (García‐Montoya *et al*., 2012). Lactoferrin is an important endogenous antimicrobial component of the innate immune system; it is principally found in tears, saliva, mucous secretions and breast milk of mammals (Cooper *et al*., 2014). Its most potent properties include sequestering iron essential for bacterial motility, and direct interaction with Gram‐negative bacterial cell walls to induce lysis at the site of infection (Valenti *et al*., 2015). This consequently means that its bacteriostatic effect can prevent biofilm development, but also disrupt cells which have already become adherent. Lactoferrin has the additional benefit of exhibiting anti‐inflammatory properties, which may play an important role in mitigating chronic inflammation associated with delayed wound healing (Valenti *et al*., 2017). Allison *et al*. (2015) showed that as a component of breast milk, lactoferrin at a concentration of 3 mg ml^−1^ significantly decreased S*treptococcus mutans* biofilm formation *in vitro. *The artificial sweetener xylitol has been shown to bind to Gram‐positive organisms, preventing the organism from adhering to other cells or surfaces (Ferreira *et al*., 2015). The co‐administration of lactoferrin and xylitol has shown great efficacy in eliminating *Pseudomonas aeruginosa* biofilms *in vitro* (Ammons *et al*., 2011a,2011b). The acquisition of iron by lactoferrin causes membrane disruption, whilst xylitol prevents *P. aeruginosa* biofilms successfully responding to the environmental change (Rhoads *et al*., 2008). This treatment modality has been successfully implemented for the treatment of chronic wounds; a lactoferrin and xylitol hydrogel, in conjunction with a silver wound dressing, were shown to achieve a significant log reduction in *Staphylococcus aureus* and *P. aeruginosa* biofilm viability of 6.5 ± 2.4 and 4.9 ± 0.9 respectively (Ammons *et al*., 2011a,2011b). However, this method is comparably only as efficacious as sharp debridement and still requires treatment over a number of weeks to months, but may provide a means to prevent recalcitrant and exacerbated infection (Kim *et al*., 2018). By avoiding mechanisms of microbial resistance, quorum sensing inhibition, bacteriostasis and adhesion prevention are pertinent alternatives to traditional antimicrobial therapies. To meet the multifactorial challenge presented by biofilms in chronic wounds, this review proposes that gas‐filled microbubbles (MBs) can be the versatile biomedical tool required.

## Gas microbubbles: a method of controlled drug delivery

**Figure 2:**
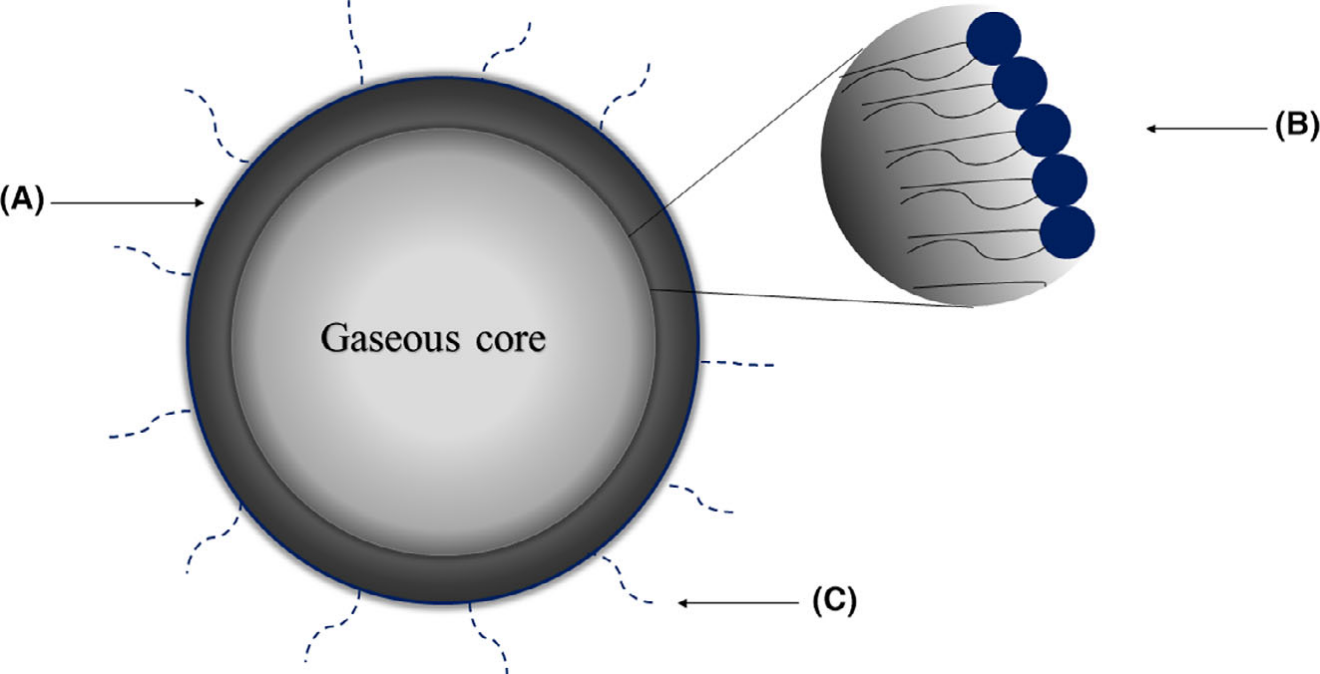
A schematic representation of a gas microbubble depicting a gaseous core encapsulated by (A) stabilizing lipid shell. An expanded view (B) shows the orientation of the phospholipid monolayer at the gas–liquid interface, with polar heads in contact with the aqueous phase and polar tails internalized towards the gaseous core. The addition of polyethylene glycol surfactant chains is represented by (C).

The architecture of gas‐filled microbubbles is variable according to their intended application, though they commonly consist of a surfactant, polymer, protein or phospholipid shell, which encapsulates a gaseous core (Fig. 2) (Sirsi and Borden, 2009; Owen *et al*., 2018). The composition of the MB shell is integral to conferring mechanical stability, preventing coalescence and determining its acoustic response to stimulation by ultrasound (US) (Borden *et al*., 2005; Stride, 2008; Carugo *et al*., 2017). Characterizing MB size is an important step for determining not only its acoustic response and drug loading capacity, but also its longevity in circulation and thereby its safety for *in vivo* applications (Lee *et al*., 2015b). MBs are typically manufactured with a diameter distribution in the range between 1 and 10 μm, and the mean MB diameter during storage increases over time (Ferrara *et al*., 2007). Notably, there exists a pressure difference between the inside of a MB and the outer environment (known as Laplace pressure), which is caused by the surface tension of the curved gas–liquid interface. For a given MB shell formulation, the Laplace pressure is inversely proportional to MB radius. Therefore, gas diffuses from the smaller bubbles to the larger ones, leading to dissolution and disappearance of the smaller bubbles. It is well documented that factors such as shell composition, fabrication method used, the relative chemical environment and temperature can substantially influence MB size and its temporal evolution (Mulvana *et al*., 2010; Sun *et al*., 2014; Lee *et al*., 2015b; Taylor *et al*., 2017).

Phospholipids are perhaps the most common excipients of the MB shell; key examples include distearoylphosphatidylcholine (DSPC) and dipalmitoylphosphatidylcholine (DPPC) (Swanson *et al*., 2010). The lipid molecules assemble in a monolayer at the hydrophilic–hydrophobic interface to stabilize the gas core, exposing their hydrophilic polar head to the aqueous environment and their hydrophobic tails towards the gaseous core (Fig. 2). The acyl chain length of a lipid dictates the permeability of the MB shell to gases (Borden, 2016); longer hydrophobic acyl chains provide greater cohesion (or packing density) between adjacent lipids (Hosny *et al*., 2013), consequently reducing MB shell permeability to gases and increasing its stability during storage and upon administration (Zhuang *et al*., 2016). Surfactants can be integrated into the MB shell, which is particularly important for biomedical applications of MBs such as drug delivery (Abou‐Saleh *et al*., 2014). The addition of the surfactant polyethylene glycol (PEG) to a MB shell simultaneously provides the means to functionalize the MB shell with biological components or assemblies such as targeting ligands, antibodies and liposomes (Abou‐Saleh *et al*., 2014), whilst mitigating immunogenicity to lipid antigens and preventing phagocytosis of the MB by macrophages (Paolino *et al*., 2017). The content of the MB gaseous core is arguably just as important as the composition of the encapsulating shell, as it dictates some of the MB properties (Vohra and Jasuja, 2016). High molecular weight perfluorocarbon compounds and sulfur hexafluoride are commonly used as the gaseous core for MBs *in vivo*; the poor water solubility and low diffusion rate of fluorinated gases prolong MB longevity by enhancing stability (Casini *et al*., 2016; Carugo *et al*., 2017). The drug loading capacity of a MB is strongly linked to the efficacy of the treatment, as it directly impacts the amount of a given therapeutic agent that can be delivered to a target site (Tzu‐Yin *et al*., 2013). The use of electrostatic force to bond drugs to the MB surface (Rychak and Klibanov, 2014), insertion into the MB shell (Lentacker *et al*., 2009), loading the drug into the gaseous void and placing a layer of oil at the interface between the gaseous core and MB shell (Tinkov *et al*., 2009) are all considered low‐capacity methods of drug loading (Sirsi and Borden, 2009). To yield a greater MB drug loading capacity, it is common for nanoparticles or liposomes to be conjugated to MBs covalently or with the use of biotin–avidin bridges (Lentacker *et al*., 2010; Liang *et al*., 2018). However, methods of achieving a greater drug loading capacity also directly impact upon the stability and acoustic properties of the MB, due to their altered shell thickness and composition (Tzu‐Yin *et al*., 2013; Kooiman *et al*., 2014). Drug loading modalities, methods of microbubble fabrication and their biophysical effects have been extensively reviewed elsewhere (Unger *et al*., 2002; Stride and Edirisinghe, 2008; Fix *et al*., 2015; Lee *et al*., 2015b).

## Ultrasound‐mediated physical effects of gas microbubbles

Manipulation of the MB fabrication method and shell composition dictates their physico‐chemical properties and size, allowing them to elicit different modes of action in response to acoustic stimulation. These ultrasound‐mediated behaviours of the MBs can be further regulated by adjusting the parameters of the US delivered (e.g. frequency, acoustic pressure, duration, pulse repetition frequency); this makes MBs perhaps one of the most versatile tools available in biomedicine. In biomedical applications of MBs, their efficacy for a given task is correlated to their acoustic response (Datta *et al*., 2006; Choi *et al*., 2014), which is typically categorized into either stable or inertial cavitation (Lentacker *et al*., 2014). In response to the pressure changes of ultrasound waves, MBs experience alternating volumetric compression and expansion (or rarefaction) (De Jong *et al*., 2002). Upon exposure to low‐intensity US, the nature of these oscillations is typically repetitive over several US cycles; this behaviour is referred to as stable cavitation (Stride and Coussios, 2009). Above a critical US intensity, the periodicity of this oscillation is lost; MBs expand rapidly, and the inertia of the surrounding fluid during contraction causes them to collapse violently (Wu and Nyborg, 2008; Stride and Coussios, 2009). This process is known as inertial or transient cavitation and often leads to MB fragmentation into smaller bubbles. It should be noted that whilst there is a simple relationship between US intensity and pressure for a plane travelling wave, it is more complex in 2D fields and standing wave fields. The propensity for fragmentation of lipid‐shelled MBs is inversely correlated to the phospholipid alkyl chain length and also depends upon the type of emulsifier used (Borden *et al*., 2005). Notably, the MB resonant frequency and amplitude of oscillation, the transition pressure from stable to inertial cavitation, and MB fragmentation diameter all strongly depend upon the initial MB size (for a given shell formulation and suspension medium) (Povey *et al*., 1998; Borden *et al*., 2005). Therefore, the selection of a specific US frequency to elicit a desired acoustic response should take into account the typically broad size distribution of a MB suspension and variations in MB size during sample storage and/or handling.

**Figure 3:**
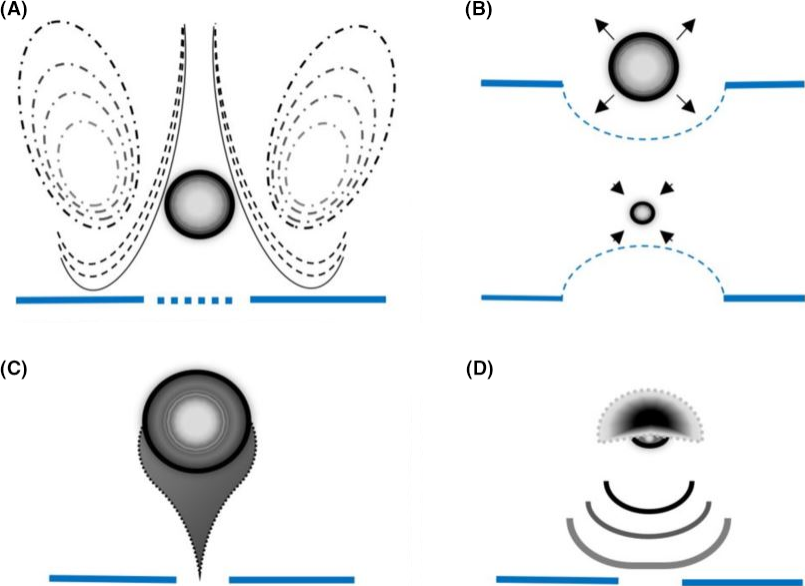
Examples of the biophysical effects of microbubbles on cell membranes (**––––**), when exposed to varied ultrasound parameters: (A) microstreaming of fluid is generated upon temporally sustained oscillation of the microbubble; the mechanical stress imparted on proximal cell membranes can lead to pore formation. (B) microbubble pushing upon expansion and pulling upon compression are characteristic effects of stable cavitation; this can increase cell membrane permeability and/or compromise membrane integrity. (C) if a microbubble undergoes inertial cavitation near a surface, the resulting collapse of the microbubble is asymmetrical and leads to the generation of a liquid microjet directed towards the surface. Fluid jetting can cause membrane perforation and thus enhance intracellular transport of therapeutic compounds. (D) in addition to liquid jetting, shock waves can be produced by microbubbles that collapse forcefully. The stress imparted on a cell membrane can equally cause membrane perforation.

Concerning the biophysical effects of MBs, it has been postulated that the systematic expansion and compression of MBs in stable cavitation create localized pushing and pulling forces, which in turn cause disruption to the integrity of cell membranes located in their vicinity (Fig. 3B) (Lee *et al*., 2015a). It is also possible for MB oscillation to drive a steady flow of the surrounding fluid (also known as cavitation microstreaming). The volumetric oscillation of MBs generates flows that are purely divergent (i.e. radial), whilst interaction with a dissimilar surface (e.g. a target tissue) can generate a circulatory flow that enhances shear stress over nearby cells, potentially causing transmembrane pores to form (Fig. 3A) (Ferrara *et al*., 2007). Furthermore, the streaming flow field can drive shedding of shell constituents – such as therapeutic compounds – away from a MB (Luan *et al*., 2014), which in turn can be exploited as a mechanism to deposit (or ‘print’) therapeutic material over the membrane of target cells (De Cock *et al*., 2016). Microjet formation and shockwaves are more transient physical effects attributed to the collapse of MBs in inertial cavitation, which respectively puncture proximal membranes and increase membrane permeability through mechanical stress (Fig. 3C and D) (Collis *et al*., 2010). Although inertial cavitation can release energy in the form of heat, this is rapidly dissipated in the surrounding fluid, which has a significantly greater total volume than the volume occupied by MBs (Ye *et al*., 2013). In comparison with the mechanical stresses imparted by inertial cavitation, it could be inferred that stable cavitation is a comparatively gentle means of facilitating drug uptake.

## The use of ultrasound alone as an anti‐biofilm treatment

The ability of low‐frequency US to improve the efficacy of antibiotics was shown as early as 1994; the minimum inhibitory concentration (MIC) of aminoglycoside and macrolide antibiotics was consistently reduced by up to 50%, in planktonic cultures of *P. aeruginosa* and *S. aureus* exposed to continuous US at 67 kHz and 0.3 W cm^−2^ intensity (Pitt *et al*., 1994). This was later supported in a study by Runyan *et al*. (2006), who demonstrated that US both potentiates antibiotics against planktonic cultures and biofilms by facilitating the transport of antibiotics into biofilms. The prevailing theory is that US increases the permeability of cell membranes to systemically available antibiotics, without physically disrupting or dispersing the biofilm (Mohammad *et al*., 2015). The efficacy of US as an adjuvant to antibiotics can be supported by a study conducted by Pitt and Ross (2003), which showed that in the absence of antibiotic low‐frequency US (70 kHz) of < 2 W cm^−2^ acoustic intensity, increased the growth rate of planktonic *Escherichia coli* and *P. aeruginosa*. It can be reliably deduced that by the same means, US potentiates antibiotics by promoting transport across cell membranes; in the absence of antibiotics, US may increase the rate at which waste products are removed and metabolites are transported to cells, consequently enhancing their growth.

Investigations conducted *in vivo* on the effect of adjuvant US on biofilms implanted subcutaneously in rabbits showed that after 24 h of continuous US exposure (28.48‐kHz, 100 and 300 mW cm^−2^), recovered *E. coli* colony‐forming units (CFU) were significantly reduced, whereas there was no observable effect on *P. aeruginosa* (Rediske *et al*., 1999). The literature in this area is clear that US alone has no effect on cell viability, though they do lack congruency in the US frequency, intensity, and pulse length or repetition frequency used (Jiang *et al*., 2016; Cai *et al*., 2017). The principal limitation of this approach is that it currently lacks translation to a viable clinical application; the most efficacious low‐frequency US (28.48 kHz) and intensity (100 and 300 mW cm^−2^) used *in vitro* were shown to induce tissue damage when applied *in vivo* (Rediske *et al*., 1999; Jiang *et al*., 2016). It should also be noted that the majority of studies have continuous treatment times of 24‐48 h, which is unlikely to be considered economically or socially practical. Moreover, this method of utilizing the synergistic relationship between US and antibiotic efficacy does not allow for targeted or controlled delivery of the antibiotic to a localized region, since it relies on the traditional oral or intravenous administration of antibiotics of sufficient dose to ensure an effective serum concentration. The limitation of this method is that any antibiotic administered in this manner would still have a non‐specific impact on unintended systemic targets, which would include dysbiosis of the host commensal microbiota (Carding *et al*., 2015). Due to the localized release of antibiotics conjugated with microbubbles, the dosage required to achieve an effective local antibiotic concentration is significantly lower than orally or intravenously administered antibiotic (Horsley *et al*., 2019). In quantitative terms, the typical recommended dosage for systemic administration of aminoglycoside and β‐lactam antibiotics ranges from 2 to 16 g day^−1^ (Taccone *et al*., 2011), whilst the concentration associated with microbubble administration is typically of MIC (i.e. μg ml^−1^) (Pitt *et al*., 2004; Zhu *et al*., 2014). Therefore, the residual serum concentration of antibiotics delivered by microbubbles is much lower, likely reducing or removing non‐specific systemic targets.

The application of low‐frequency (20‐60 kHz) ultrasound for chronic wound debridement has shown great potential, with recent advancements extensively reviewed elsewhere (Chang *et al*., 2017; Liu *et al*., 2017). To summarize, ultrasonic debridement has been shown to potentiate antibiotics against bacteria within the chronic wound, emulsify dead cells within a localized area and stimulate peripheral healthy cells to promote the healing process. The efficacy of this treatment modality has been assessed in a number of clinical trials, which generally concur that ultrasonic debridement is a valuable wound care adjuvant (Amini *et al*., 2013; Murphy *et al*., 2018). It is important to note that although these trials have demonstrated that ultrasonic debridement improves short‐term treatment outcomes, the frequency and total duration of the treatment are comparable to non‐surgical sharp debridement with no significant difference in healing rate between the modalities after 6 months of treatment (Amini *et al*., 2013; Michailidis *et al*., 2018).

## Acoustically activated gas microbubbles for the treatment of bacterial biofilms

The success of low‐frequency US (20–100 kHz) in facilitating the uptake of systemic antibiotics by biofilms has been variable; therefore, the use of US‐responsive MBs in the light of their controllable physical response may be viewed as an evolution in antimicrobial drug delivery. Alteration in the permeability of biofilms to macromolecules, such as antimicrobial compounds, has been routinely demonstrated with the use of the red‐fluorescent nucleic acid stain, propidium iodide (PI). Dong *et al*. (2017) demonstrated that acoustically activated MBs were capable of enhancing the permeability of *Staphylococcus epidermidis* biofilms grown in OptiCell^™^ chambers *in vitro*. The fluorescent signal emitted increases proportionally with the quantity of PI that has intercalated with DNA; therefore, it can be utilized as a direct means of assessing how MBs facilitate uptake of macromolecules (Stiefel *et al*., 2015). It could be therefore hypothesized that US‐activated MBs facilitate the uptake of antibiotics by biofilms, principally through increasing membrane permeability of bacterial cells and *via* heterogeneous alterations to the biofilm architecture, which can include the development of pores in the EPS matrix (Dong *et al*., 2013; Jang *et al*., 2017; Hu *et al*., 2018). Fluid shear stress has also been shown to significantly affect biofilm morphology; at shear stresses under 1 Pa, biofilms are shown to grow in a laboratory‐typical mushroom‐like shape with interstitial channels and voids (Salta *et al*., 2013). However, when the shear stress overcomes the biofilm adhesion strength (> 2 Pa), erosion and sloughing of the biofilm are more pronounced, which leads to low‐density biofilm aggregates with large interspaces (Rmaile *et al*., 2014). Shear stress in terms of human physiology is variable according to location; arteries are typically subject to average wall shear stress of 1–3 Pa, whilst in arterioles and capillaries it is commonly of 2‐6 Pa (Sheikh *et al*., 2003; Shaik *et al*., 2009). The effect of fluid shear stress on the detachment of biofilms has shown to be compounded in the presence of microbubbles, clearing up to 70% of biofilm in a species‐dependent manner *in vitro* (Sharma *et al*., 2005). It has been shown that the *in vitro* application of 0.08 MHz, 1.0 W cm^−2^, 50% duty cycle and 10 min duration US, MBs and vancomycin can significantly decrease the number of viable cells (7.17 log_10_ CFU ml^−1^) from *S. epidermidis* biofilms compared to an untreated control (10.51 log_10_ CFU ml^−1^) (He *et al*., 2011). It is important to note, however, that this study could not demonstrate a significant difference between these groups using an *in vivo* rabbit model, in which *S. epidermidis* biofilms grown on polyethylene discs were subcutaneously implanted bilateral to the vertebral column. The work carried out by He *et al*. (2011) demonstrated that US‐activated sulfur hexafluoride MBs with a mean diameter of 2.5 μm and vancomycin create micropores within the biofilm architecture, which does support the hypothesis of facilitated uptake mediated by membrane/EPS disruption. An interesting point is that not only does the membrane disruption facilitate antibiotic uptake, but the influx of nutrients may induce a phase of active growing in deeper layers of the biofilm, potentiating the efficacy of the antibiotic (Dong *et al*., 2013). In addition, studies have consistently shown that the combination of US and MBs is capable of halving the MIC of the administered antibiotic (Kasimanickam *et al*., 2013). This has been supported in recent work by Horsley *et al*. (2019) in which gentamicin‐loaded liposomes bound to ultrasound‐responsive microbubbles were utilized to significantly enhance direct antibiotic delivery to intracellular uropathogenic bacteria. The ultrasound‐mediated delivery of gentamicin in concentrations of 0.53–1.32 μg ml^−1^ with a 20 s exposure time showed an equivalent efficacy in bacterial killing to a 2 h exposure to free gentamicin at the significantly higher clinically approved dosage of 200 μg ml^−1^ (Horsley *et al*., 2019). Moreover, the ultrasound‐mediated therapy achieved a 75% greater reduction in bacterial bioburden than free gentamicin alone, with no evidence of cellular damage (Horsley *et al*., 2019). This effect is perhaps indicative of the mechanical action of the oscillating microbubble, aiding the physical detachment of bacteria from proximal surfaces. This work has exemplified the utility of ultrasound‐mediated intracellular delivery of antimicrobial agents, as a viable alternative to the use of orally administered antibiotics. It should not be overlooked that to date, research on enhancing efficacy of US‐activated microbubbles has only been performed on naive single‐species biofilm models. Furthermore, although the biophysical effect of acoustically activated MBs is evident, the underlying mechanisms of interaction between the bubbles and the biofilm have not been elucidated yet.

## The applications of nitric oxide for the treatment of bacterial biofilms

Nitric oxide (NO) has been utilized to facilitate healing of chronic wounds such as DFU for a number of years; this is motivated by the role of NO as an important biological signalling molecule (Witte and Barbul, 2002). Cellular proliferation, angiogenesis and remodelling are key biological and physiological processes mediated by NO, which have been principally applied to wounds in the form of inducible NO synthase (Dhall *et al*., 2014). It is important to note that traditionally NO has only been applied to chronic wounds in the context of tissue repair, and not with the specific intention to treat the underlying biofilm (Saidkhani *et al*., 2016; Han and Ceilley, 2017). The administration of NO in a therapeutic capacity has historically been difficult; at high concentrations, NO is bactericidal and cytotoxic, which significantly impairs the progression of the normal healing process (Schulz and Stechmiller, 2006). Studies of the biofilm life cycle have elucidated endogenous mechanisms which can be exploited as therapeutic targets, these principally involve the use of NO in the sub‐micromolar range to induce the biofilm dispersal phase (Barraud *et al*., 2015). By inducing dispersal of the biofilm with NO, the physical barrier imposed by the EPS matrix can be negated entirely. Research has shown that the dispersed cells are considerably more susceptible to antimicrobial treatments; it can therefore be inferred that adjuvant NO potentiates antibiotics against biofilms (Howlin *et al*., 2017). Utilizing the spontaneous NO donor sodium nitroprusside (SNP), Howlin *et al*. (2017) successfully showed that NO disrupted *P. aeruginosa* biofilms from cystic fibrosis sputum samples *in vitro*. The same study also highlighted the importance of dispersal as a means of therapy, since the administration of the antibiotic tobramycin alone caused a significant increase in biomass and biofilm thickness compared to untreated controls. Xu *et al*. (2017) have proposed and tested the implementation of NO‐releasing agents for indwelling medical device surfaces, and they were able to successfully prevent biofilm formation on a functionalized polyurethane surface. Utilizing NO to prevent bacterial growth and adhesion on implanted surfaces in conjunction with antimicrobial therapy may result in better clinical outcomes for patients and significant cost savings for health service providers. There is relatively little published data either *in vivo* or *in vitro,* on the successful implementation of exogenous NO in a gaseous form for biofilm dispersal. The inhalation of NO has been an FDA‐approved therapeutic agent for nearly two decades, and clinical trials have shown that NO gas at 5–10 ppm can achieve a 3.5 log reduction in respiratory *P. aeruginosa* biofilm aggregates (Howlin *et al*., 2017). The major problem with the delivery of NO in a gaseous form is that it is highly reactive, which translates to a half‐life of only seconds (Thomas *et al*., 2001). A solution to this challenge is to utilize an US‐responsive agent such as the MB, which could provide the means to successfully control the delivery and release of NO.

## The mechanism of action for nitric oxide‐induced dispersal of biofilms

The control of dispersal events in the biofilm life cycle is linked to the intracellular second messenger molecule cyclic‐di‐guanosine monophosphate (c‐di‐GMP), which is regulated by cellular phosphodiesterase (Reinders *et al*., 2016). Recent studies have shown that the activity of cellular phosphodiesterase is increased in the presence of NO at concentrations in the pico‐ and nanomolar range, which results in the degradation of c‐di‐GMP and subsequent dispersal of the biofilm (Algburi *et al*., 2017; Howlin *et al*., 2017). The dispersed cells return to a motile state and become susceptible to antimicrobial treatment; this effect is shown to be conserved across species such as *P. aeruginosa and Staphylococcus aureus* and in both single and complex multispecies biofilms (Barraud *et al*., 2009). Dispersal has been achieved with NO concentrations as low as 450 pM, reaching peak efficacy at 450 nM (Howlin *et al*., 2017).

## Biologically active nitric oxide gas microbubbles and their applications

**Figure 4:**
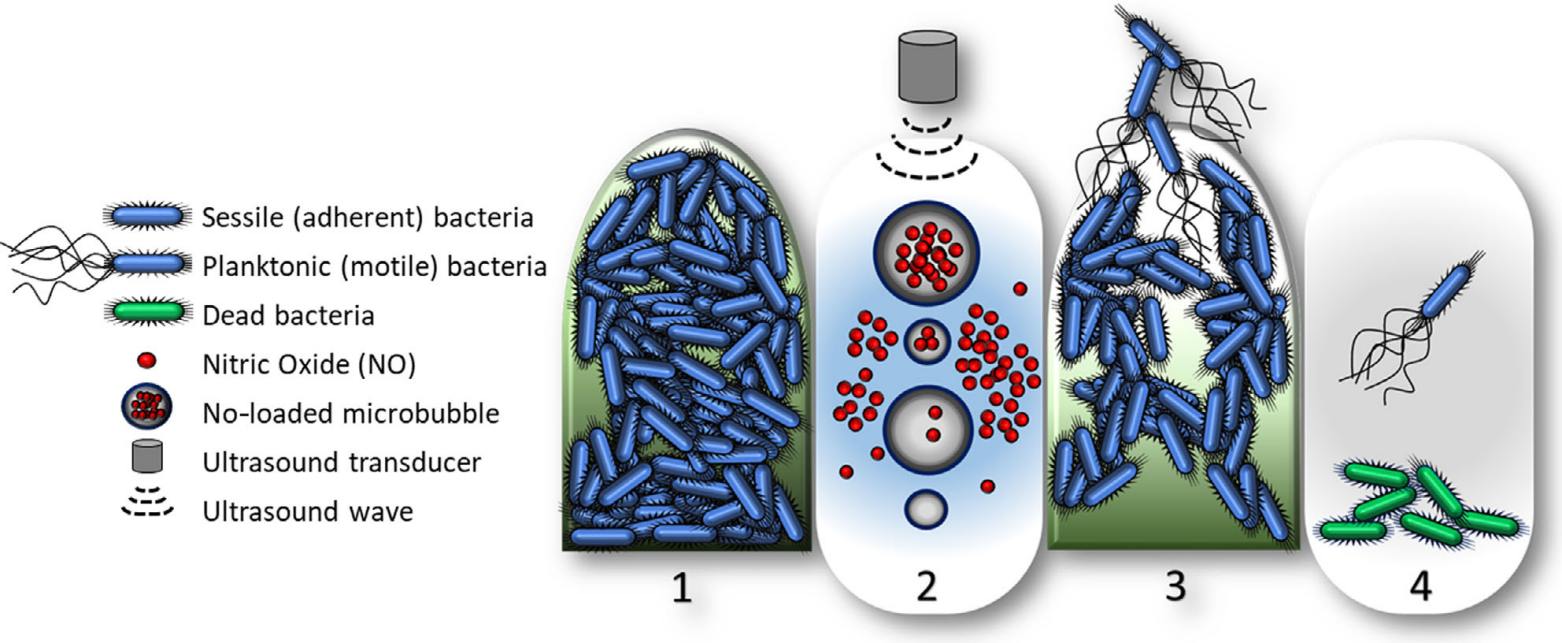
Gas microbubbles undergoing stable cavitation in response to ultrasound have been shown to enhance membrane permeability; this can increase the local intracellular concentration of a target drug. By encapsulating biologically active nitric oxide as the gaseous core of a shelled microbubble, the combined mechanical action of the oscillating microbubble and biological effect of nitric oxide may induce targeted dispersal and elimination of biofilms from a surface. The co‐administration of antibiotic and microbubbles may provide a novel means of combating biofilm‐associated antibiotic tolerance.

Though high molecular weight gases are most commonly used as the MB core constituent, it is also possible to generate MBs that possess a biologically active gaseous core. Recent research has already highlighted the advantages of using MBs with an oxygen core, to increase the therapeutic efficacy of chemo‐sonodynamic therapy (SDT) in the targeted treatment of solid malignant tumours (McEwan *et al*., 2015; Nesbitt *et al*., 2018). Bioactive gases such as NO have significant therapeutic potential, as they mediate a number of important biological signal pathways (Basudhar *et al*., 2016). However, the molecule is highly reactive with both exogenous molecules, such as oxygen, and endogenous scavengers such as haemoglobin (Azarov *et al*., 2005). This can be mitigated by utilizing the gas as the core of acoustically active MBs; protected by the MB shell, the release of NO can then be both spatially and temporally controlled by US (Fig. 4) (Fix *et al*., 2015). The stable expansion and contraction of the MB are an important attribute of its associated biophysical effects*;* however, the effects of cavitation‐enhanced gas exchange have been less investigated. During MB compression, there is an efflux of core gas into the local environment, followed by a net influx of gas upon expansion (Crum, 1984; Lentacker *et al*., 2014; Yusof *et al*., 2016). This is particularly important in regard to the use of nitric oxide microbubbles (NOMBs), since it shows that the MB has the capacity to deliver a locally concentrated therapeutic NO payload.

There have been few studies to date, which investigated the use of microbubbles for the delivery of NO. Tong *et al*. (2013) and Wang *et al*. (2013) generated nitric oxide microbubbles (NOMBs) with a mean diameter of 3.85 μm by continuous sonication of a lipid suspension at 100 W. The organophosphorus compound 1,2‐bis(diphenylphosphino)ethane (DPPE), PEG_2000_ and phosphatidylcholine were used to encapsulate NO, which was administered at a constant stream of 4 ml min^−1^ for 5 min under anoxic conditions. In contrast, Grishenkov *et al*. (2015) and Cavalieri *et al*. (2008), used biocompatible polyvinyl alcohol (PVA) and high shear stirring to fabricate air‐filled MBs, which were then freeze‐dried with liquid nitrogen. The subsequent 4 μm diameter PVA capsules were enclosed in a pressure chamber purged with nitrogen, before loading with NO and re‐suspend in water. All four studies focused on the intravascular application of NOMBs; thus, their research is linked by some common themes.

The work by Cavalieri *et al*. (2008) was the first *in vitro* study of its kind, utilizing NOMBs for localized delivery of NO for the prevention of clot formation. Similarly, the NOMBs developed by Grishenkov *et al*. were implemented in a theranostic capacity for myocardial ischaemia, showing that they are a highly effective tool for the localized and targeted delivery of NO. Unlike the work by Tong *et al*. (2013), this study sought to use NOMBs as a prophylaxis for patients at risk of thromboembolism. In a rodent model of myocardial infarction, 60 s of continuous US (1 MHz, 1 W cm^−2^) applied to NOMBs in conjunction with mesenchymal stem cells was successfully used to promote angiogenesis (Tong *et al*., 2013). The applications of this as a successful therapy are promising, with the scope to prevent heart failure by restoring adequate blood flow to damaged cardiac tissue (Cochain *et al*., 2013). The use of NOMBs without ultrasound for the resolution of deep vein thrombosis was investigated by Wang *et al*. (2013), who successfully showed a reduction in thrombus size by 40% and mitigated development of chronic inflammation.

The research conducted by Grishenkov *et al*. (2015) was the only study to assess the final NOMB gas content and dissolution rate in solution, with and without the application of US (1–15 MHz, < 100 kPa). High‐performance liquid chromatography (HPLC) was used to measure nitrite and nitrate in both aerated and degassed saline, which are oxidized products of NO. Passive release of NO from degassed saline occurred in 17 min, and this is increased to 55 min in aerated saline; the application of US reduced the exponential time constant to 10 min and 4 min respectively (Grishenkov *et al*., 2015). The assessment of nitrate and nitrite in solution is concordant with expected parameters of diffusion; NO enters the degassed solution at a faster rate than an aerated solution.

## Multifunctional agents for the delivery of nitric oxide to biofilms: present and future perspectives

To the best of the authors’ knowledge, only one previous study has investigated the use of a NO‐releasing particle for the treatment of biofilms. The research carried out by Hetrick *et al*. (2009) investigated the use of NO‐releasing silica nanoparticles; however, it focused only on the bactericidal efficacy of the NO and not on biofilm dispersal. Consequently, there are a number of unexplored and novel aspects in this area, pertaining specifically to the application of NOMBs for the treatment of bacterial biofilms. There are currently no acoustically stimulated NOMBs used for the treatment of biofilms *in vitro* or *in vivo*; consequently, there is currently no evidence on the efficacy of NOMBs or nanoparticle‐induced dispersal of biofilms. Furthermore, though its effects have been observed, the specific interaction between MBs and the biofilm has not been fully elucidated. Previous research has shown that lipid transfer occurs between MBs and biological membranes, which undoubtedly impacts upon cellular integrity, permeability and signalling (Carugo *et al*., 2017). This effect could be successfully employed to exert a priming effect upon biofilms, to stimulate disruption of the biofilm prior to the administration of US.

Bacterial infection and subsequent development of biofilms in open wounds pose a significant risk to human health. Due to the increased tolerance of biofilms to traditional antimicrobial therapies and non‐specific drug delivery, interdisciplinary techniques are being explored as novel treatment methods. Ultrasound‐responsive drug delivery agents provide a dynamic means of delivering therapeutic compounds, with high temporal and spatial specificity. Ongoing research has shown that ultrasound‐responsive agents can facilitate drug delivery, utilizing both bioactive components and mechanical stimulation to eliminate biofilms. There is no clinically viable translation of this treatment modality for chronic wounds at present, perhaps due to the complexity of ensuring consistent and efficacious implementation with minimal training or expertise. Essential parameters such as the consistent production and administration of MBs, controlled transmission of US and handling of biohazardous residuum would require a robust system with clearly defined instructions for use in lay terms. Moreover, due to the lack of congruity in size, shape and depth of wounds, it is likely a successful clinical translation would be primarily targeted at early stages of DFU development for patients presenting with small lesions. This would then have the potential to either prevent or limit biofilm development, in addition to dispersing and treating any adherent cells already present in the wound bed. The transmission of US *via* a fluid stream to biotic and abiotic surfaces for the purpose of biological decontamination has recently demonstrated that clinical translation and utility in this field are achievable (Birkin *et al*., 2015, 2016). In order to achieve fundamental change in healthcare practices such as the treatment of chronic wounds, we believe this review exemplifies the need for collaborative and interdisciplinary research to potentiate existing therapies and develop novel treatment modalities.

## Conflict of interest

None declared.

## References

[mbt213471-bib-0001] Abou‐Saleh, R.H. , Swain, M. , Evans, S.D. , and Thomson, N.H. (2014) Poly(ethylene glycol) lipid‐shelled microbubbles: abundance, stability, and mechanical properties. *Langmuir* Am Chem Soc 30: 5557–5563. 10.1021/la404804u. 24758714

[mbt213471-bib-0002] Algburi, A. , Comito, N. , Kashtanov, D. , Dicks, L.M.T. , and Chikindas, M.L. (2017) Control of biofilm formation: antibiotics and beyond. Appl Environ Microbiol 83: e02508‐16 10.1128/AEM.02508-16. 27864170 PMC5244297

[mbt213471-bib-0003] Allison, L. , Walker, L. , Sanders, B. , Yang, Z. , Eckert, G. , and Gregory, R. (2015) Effect of human milk and its components on *Streptococcus mutans* biofilm formation. J Clin Pediatr Dent 39: 255–261. 10.17796/1053-4628-39.3.255. 26208071

[mbt213471-bib-0004] Amini, S. , ShojaeeFard, A. , Annabestani, Z. , Hammami, M.R. , Shaiganmehr, Z. , Larijani, B. , *et al* (2013) Low‐frequency ultrasound debridement in patients with diabetic foot ulcers and osteomyelitis. Wounds 25: 193–198. 25867038

[mbt213471-bib-0005] Ammons, M.C.B. , Ward, L.S. , Dowd, S. , and James, G.A. (2011a) Combined treatment of *Pseudomonas aeruginosa* biofilm with lactoferrin and xylitol inhibits the ability of bacteria to respond to damage resulting from lactoferrin iron chelation. Int J Antimicrob Agents 37: 316–323. 10.1016/j.ijantimicag.2010.12.019. 21377840 PMC7008007

[mbt213471-bib-0006] Ammons, M.C.B. , Ward, L.S. and James, G.A. (2011b) Anti‐biofilm efficacy of a lactoferrin/xylitol wound hydrogel used in combination with silver wound dressings. Int Wound J 8: 268–273. 10.1111/j.1742-481X.2011.00781.x 21457463 PMC7050428

[mbt213471-bib-0007] Attinger, C. and Wolcott, R. (2012) Clinically addressing biofilm in chronic wounds. Adv Wound Care 1: 127–132. 10.1089/wound.2011.0333 PMC3839004 24527292

[mbt213471-bib-0008] Azarov, I. , Huang, K.T. , Basu, S. , Gladwin, M.T. , Hogg, N. , and Kim‐Shapiro, D.B. (2005) Nitric oxide scavenging by red blood cells as a function of hematocrit and oxygenation. J Biol Chem 280: 39024–39032. 10.1074/jbc.M509045200. 16186121

[mbt213471-bib-0009] Banu, A. , Noorul Hassan, M.M. , Rajkumar, J. , and Srinivasa, S. (2015) Spectrum of bacteria associated with diabetic foot ulcer and biofilm formation: a prospective study. Aust Med J 8: 280–285. 10.4066/AMJ.2015.2422. PMC4592943 26464584

[mbt213471-bib-0010] Barraud, N. , Storey, M.V. , Moore, Z.P. , Webb, J.S. , Rice, S.A. and Kjelleberg, S. (2009) Nitric oxide‐mediated dispersal in single‐ and multi‐species biofilms of clinically and industrially relevant microorganisms. Microb Biotechnol 2: 370–378. 10.1111/j.1751-7915.2009.00098.x 21261931 PMC3815757

[mbt213471-bib-0011] Barraud, N. , Kelso, M.J. , Rice, S.A. , and Kjelleberg, S. (2015) Nitric oxide: a key mediator of biofilm dispersal with applications in infectious diseases. Curr Pharmaceut Des 21: 31–42. 10.2174/1381612820666140905112822 25189865

[mbt213471-bib-0012] Bartell, J.A. , Sommer, L.M. , Haagensen, J.A.J. , Loch, A. , Espinosa, R. , Molin, S. , and Johansen, H.K. (2019) Evolutionary highways to persistent bacterial infection. Nature Communications 10: 629 10.1038/s41467-019-08504-7. PMC6367392 30733448

[mbt213471-bib-0013] Basudhar, D. , Ridnour, L.A. , Cheng, R. , Kesarwala, A.H. , Heinecke, J. , and Wink, D.A. (2016) Biological signaling by small inorganic molecules. Coord Chem Rev 306: 708–723. 10.1016/J.CCR.2015.06.001. 26688591 PMC4680994

[mbt213471-bib-0014] Birkin, P.R. , Offin, D.G. , Vian, C.J.B. , Howlin, R.P. , Dawson, J.I. , Secker, T.J. , *et al* (2015) Cold water cleaning of brain proteins, biofilm and bone – harnessing an ultrasonically activated stream. Phys Chem Chem Phys 17: 20574–20579. 10.1039/C5CP02406D. 26200694

[mbt213471-bib-0015] Birkin, P.R. , Offin, D.G. and Leighton, T.G. (2016) An activated fluid stream – New techniques for cold water cleaning. 10.1016/j.ultsonch.2015.10.001 26522990

[mbt213471-bib-0016] Bjarnsholt, T. (2013) The role of bacterial biofilms in chronic infections. APMIS 121: 1–58. 10.1111/apm.12099. 23635385

[mbt213471-bib-0017] Borden, M.A. (2016) Lipid‐coated nanodrops and microbubbles In Handbook of Ultrasonics and Sonochemistry. Singapore: Springer Singapore, pp. 1075–1100. 10.1007/978-981-287-278-4_26.

[mbt213471-bib-0018] Borden, M.A. , Kruse, D.E. , Caskey, C.F. , Zhao, S. , Dayton, P.A. and Ferrara, K.W. (2005) Influence of lipid shell physicochemical properties on ultrasound‐induced microbubble destruction. IEEE Trans Ultrason Ferroelectr Freq Control 52: 1992–2002. 16422411 10.1109/tuffc.2005.1561668 PMC1388091

[mbt213471-bib-0019] Cai, Y. , Wang, J. , Liu, X. , Wang, R. , and Xia, L. (2017) A review of the combination therapy of low frequency ultrasound with antibiotics. Biomed Res Int 2017: 1–14. 10.1155/2017/2317846. PMC5662814 29124063

[mbt213471-bib-0020] Cardinal, M. , Eisenbud, D.E. , Armstrong, D.G. , Zelen, C. , Driver, V. , Attinger, C. , *et al* (2009) Serial surgical debridement: a retrospective study on clinical outcomes in chronic lower extremity wounds. Wound Repair Regen 17: 306–311. 10.1111/j.1524-475X.2009.00485.x. 19660037

[mbt213471-bib-0021] Carding, S. , Verbeke, K. , Vipond, D.T. , Corfe, B.M. and Owen, L.J. (2015) Dysbiosis of the gut microbiota in disease. Microb Ecol Health Dis 26: 26191 10.3402/MEHD.V26.26191 25651997 PMC4315779

[mbt213471-bib-0022] Carugo, D. , Aron, M. , Sezgin, E. , Bernardino de la Serna, J. , Kuimova, M.K. , Eggeling, C. , and Stride, E. (2017) Modulation of the molecular arrangement in artificial and biological membranes by phospholipid‐shelled microbubbles. Biomaterials 113: 105–117. 10.1016/J.BIOMATERIALS.2016.10.034 27814482

[mbt213471-bib-0023] Casini, G. , Loiudice, P. , De Cillà, S. , Radice, P. , and Nardi, M. (2016) Sulfur hexafluoride (SF6) versus perfluoropropane (C3F8) tamponade and short term face‐down position for macular hole repair: a randomized prospective study. Int J Retina Vitreous 2: 10 10.1186/s40942-016-0036-9 27847628 PMC5088452

[mbt213471-bib-0024] Cavalieri, F. , Finelli, I. , Tortora, M. , Mozetic, P. , Chiessi, E. , Polizio, F. , *et al* (2008) Polymer microbubbles as diagnostic and therapeutic gas delivery device. Chem Mater 20: 3254–3258. 10.1021/cm703702d

[mbt213471-bib-0025] Chang, Y.‐J.R. , Perry, J. , and Cross, K. (2017) Low‐frequency ultrasound debridement in chronic wound healing: a systematic review of current evidence. Plast Surg 25: 21–26. 10.1177/2292550317693813 PMC5626185 29026808

[mbt213471-bib-0026] Choi, J.J. , Carlisle, R.C. , Coviello, C. , Seymour, L. , and Coussios, C.‐C. (2014) Non‐invasive and real‐time passive acoustic mapping of ultrasound‐mediated drug delivery. Phys Med Biol 59: 4861–4877. 10.1088/0031-9155/59/17/4861 25098262

[mbt213471-bib-0027] Chua, S.L. , Liu, Y. , Yam, J.K.H. , Chen, Y. , Vejborg, R.M. , Tan, B.G.C. , *et al* (2014) Dispersed cells represent a distinct stage in the transition from bacterial biofilm to planktonic lifestyles. Nat Commun 5: 4462 10.1038/ncomms5462 25042103

[mbt213471-bib-0028] Clark, R.A.F. (1993) Biology of dermal wound repair. Dermatol Clin 11: 647–666. 10.1016/S0733-8635(18)30218-3. 8222349

[mbt213471-bib-0029] Cochain, C. , Channon, K.M. , and Silvestre, J.‐S. (2013) Angiogenesis in the infarcted myocardium. Antioxid Redox Signal 18: 1100–1113. 10.1089/ars.2012.4849. 22870932 PMC3567783

[mbt213471-bib-0030] Collis, J. , Manasseh, R. , Liovic, P. , Tho, P. , Ooi, A. , Petkovic‐Duran, K. , and Zhu, Y. (2010) Cavitation microstreaming and stress fields created by microbubbles. Ultrasonics 50: 273–279. 10.1016/J.ULTRAS.2009.10.002. 19896683

[mbt213471-bib-0031] Cooper, R.A. , Bjarnsholt, T. , and Alhede, M. (2014) Biofilms in wounds: a review of present knowledge. J Wound Care 23: 570–582. 10.12968/jowc.2014.23.11.570. 25375405

[mbt213471-bib-0032] Costa, R.H.R. , Cardoso, N.A. , Procópio, R.J. , Navarro, T.P. , Dardik, A. , and de Loiola Cisneros, L. (2017) Diabetic foot ulcer carries high amputation and mortality rates, particularly in the presence of advanced age, peripheral artery disease and anemia. Clin Res Rev 11: S583–S587. 10.1016/J.DSX.2017.04.008. 28465149

[mbt213471-bib-0033] Crum, L.A. (1984) Acoustic cavitation series: part five rectified diffusion. Ultrasonics 22: 215–223. 10.1016/0041-624X(84)90016-7.

[mbt213471-bib-0034] Datta, S. , Coussios, C.‐C. , McAdory, L.E. , Tan, J. , Porter, T. , De Courten‐Myers, G. , and Holland, C.K. (2006) Correlation of cavitation with ultrasound enhancement of thrombolysis. Ultrasound Med Biol 32: 1257–1267. 10.1016/J.ULTRASMEDBIO.2006.04.008. 16875959 PMC1937506

[mbt213471-bib-0035] De Cock, I. , Lajoinie, G. , Versluis, M. , De Smedt, S.C. , and Lentacker, I. (2016) Sonoprinting and the importance of microbubble loading for the ultrasound mediated cellular delivery of nanoparticles. Biomaterials 83: 294–307. 10.1016/J.BIOMATERIALS.2016.01.022. 26796042

[mbt213471-bib-0036] De Jong, N. , Bouakaz, A. , and Frinking, P. (2002) Basic acoustic properties of microbubbles. Echocardiography 19: 229–240. 12022933 10.1046/j.1540-8175.2002.00229.x

[mbt213471-bib-0037] Dhall, S. , Do, D. , Garcia, M. , Wijesinghe, D.S. , Brandon, A. , Kim, J. , *et al* (2014) A novel model of chronic wounds: importance of redox imbalance and biofilm‐forming bacteria for establishment of chronicity. PLoS ONE 9: e109848 10.1371/journal.pone.0109848 25313558 PMC4196950

[mbt213471-bib-0038] Dong, Y. , Chen, S. , Wang, Z. , Peng, N. , and Yu, J. (2013) Synergy of ultrasound microbubbles and vancomycin against Staphylococcus epidermidis biofilm. J Antimicrob Chemother 68: 816–826. 10.1093/jac/dks490. 23248238

[mbt213471-bib-0039] Dong, Y. , Xu, Y. , Li, P. , Wang, C. , Cao, Y. , and Yu, J. (2017) Antibiofilm effect of ultrasound combined with microbubbles against Staphylococcus epidermidis biofilm. Int J Med Microbiol 307: 321–328. 10.1016/j.ijmm.2017.06.001. 28610835

[mbt213471-bib-0040] Donlan, R.M. (2002) Biofilms: microbial life on surfaces. Emerg Infect Dis 8: 881–890. 10.3201/eid0809.020063 12194761 PMC2732559

[mbt213471-bib-0041] Ferrara, K. , Pollard, R. , and Borden, M. (2007) Ultrasound microbubble contrast agents: fundamentals and application to gene and drug delivery. Ann Rev Biomed Eng 9: 415–447. 10.1146/annurev.bioeng.8.061505.095852. 17651012

[mbt213471-bib-0042] Ferreira, S. , Silva‐Paes‐Leme, F. , Raposo, R.B. and da Silva, S. (2015) By passing microbial resistance: xylitol controls microorganisms growth by means of its anti‐adherence property. Curr Pharm Biotechnol 16, 35–42. 25483720 10.2174/1389201015666141202104347

[mbt213471-bib-0043] Figueiredo, A.M.S. , Ferreira, F.A. , Beltrame, C.O. , and Côrtes, M.F. (2017) The role of biofilms in persistent infections and factors involved in *ica* ‐independent biofilm development and gene regulation in *Staphylococcus aureus* . Crit Rev Microbiol 43: 602–620. 10.1080/1040841X.2017.1282941. 28581360

[mbt213471-bib-0044] Fix, S.M. , Borden, M.A. , and Dayton, P.A. (2015) Therapeutic gas delivery via microbubbles and liposomes. J Control Release 209: 139–149. 10.1016/j.jconrel.2015.04.027. 25913365

[mbt213471-bib-0045] Flemming, H.‐C. , Neu, T.R. , and Wozniak, D.J. (2007) The EPS matrix: the “House of Biofilm Cells”. J Bacteriol 189: 7945–7947. 10.1128/JB.00858-07. 17675377 PMC2168682

[mbt213471-bib-0046] Flemming, H.‐C. , Wingender, J. , Szewzyk, U. , Steinberg, P. , Rice, S.A. , and Kjelleberg, S. (2016) Biofilms: an emergent form of bacterial life. Nat Rev Microbiol 14: 563–575. 10.1038/nrmicro.2016.94. 27510863

[mbt213471-bib-0047] García‐Montoya, I.A. , Cendón, T.S. , Arévalo‐Gallegos, S. and Rascón‐Cruz, Q. (2012) Lactoferrin a multiple bioactive protein: an overview. Biochimica et Biophysica Acta (BBA) – General Subjects 1820: 226–236. 10.1016/J.BBAGEN.2011.06.018 21726601 PMC7127262

[mbt213471-bib-0048] Geisinger, E. and Isberg, R.R. (2017) Interplay between antibiotic resistance and virulence during disease promoted by multidrug‐resistant bacteria. J Infect Dis 215(Suppl. 1): S9–S17. 10.1093/infdis/jiw402 28375515 PMC5853982

[mbt213471-bib-0049] Grishenkov, D. , Gonon, A. , Weitzberg, E. , Lundberg, J.O. , Harmark, J. , Cerroni, B. , *et al* (2015) Ultrasound contrast agent loaded with nitric oxide as a theranostic microdevice. Drug Design, Dev Ther 9: 2409–2419. 10.2147/DDDT.S77790. PMC4425237 25995614

[mbt213471-bib-0050] Guo, S. , and DiPietro, L.A. (2010) Factors affecting wound healing. J Dental Res 89: 219–229. 10.1177/0022034509359125. PMC2903966 20139336

[mbt213471-bib-0051] Hall, C.W. , and Mah, T.‐F. (2017) Molecular mechanisms of biofilm‐based antibiotic resistance and tolerance in pathogenic bacteria. FEMS Microbiol Rev 41: 276–301. 10.1093/femsre/fux010. 28369412

[mbt213471-bib-0052] Hall‐Stoodley, L. , Costerton, J.W. , and Stoodley, P. (2004) Bacterial biofilms: from the Natural environment to infectious diseases. Nat Rev Microbiol 2: 95–108. 10.1038/nrmicro821. 15040259

[mbt213471-bib-0053] Han, G. , and Ceilley, R. (2017) Chronic wound healing: a review of current management and treatments. Adv Ther 34: 599–610. 10.1007/s12325-017-0478-y. 28108895 PMC5350204

[mbt213471-bib-0054] He, N. , Hu, J. , Liu, H. , Zhu, T. , Huang, B. , Wang, X. , *et al* (2011) Enhancement of vancomycin activity against biofilms by using ultrasound‐targeted microbubble destruction. Antimicrob Agents Chemother 55: 5331–5337. 10.1128/AAC.00542-11. 21844319 PMC3195034

[mbt213471-bib-0055] Hengzhuang, W. , Wu, H. , Ciofu, O. , Song, Z. , and Høiby, N. (2012) *In Vivo* pharmacokinetics/pharmacodynamics of colistin and imipenem in *Pseudomonas aeruginosa* biofilm infection. Antimicrob Agents Chemother 56: 2683–2690. 10.1128/AAC.06486-11. 22354300 PMC3346607

[mbt213471-bib-0056] Hetrick, E.M. , Shin, J.H. , Paul, H.S. , and Schoenfisch, M.H. (2009) Anti‐biofilm efficacy of nitric oxide‐releasing silica nanoparticles. Biomaterials 30: 2782–2789. 10.1016/J.BIOMATERIALS.2009.01.052. 19233464 PMC2692680

[mbt213471-bib-0057] Høiby, N. , Ciofu, O. , Johansen, H.K. , Song, Z. , Moser, C. , Jensen, P.Ø. , *et al* (2011) The clinical impact of bacterial biofilms. Int J Oral Sci 3: 55–65. 10.4248/IJOS11026. 21485309 PMC3469878

[mbt213471-bib-0058] Holt, J.E. , Houston, A. , Adams, C. , Edwards, S. and Kjellerup, B.V. (2017) Role of extracellular polymeric substances in polymicrobial biofilm infections of *Staphylococcus epidermidis* and *Candida albicans* modelled in the nematode *Caenorhabditis elegans* . Pathog Dis 75: ftx052. 10.1093/femspd/ftx052 PMC6251683 28475673

[mbt213471-bib-0059] Horsley, H. , Owen, J. , Browning, R. , Carugo, D. , Malone‐Lee, J. , Stride, E. , and Rohn, J.L. (2019) Ultrasound‐activated microbubbles as a novel intracellular drug delivery system for urinary tract infection. J Control Release 301: 166–175. 10.1016/J.JCONREL.2019.03.017. 30904501

[mbt213471-bib-0060] Hosny, N.A. , Mohamedi, G. , Rademeyer, P. , Owen, J. , Wu, Y. , Tang, M.‐X. , *et al* (2013) Mapping microbubble viscosity using fluorescence lifetime imaging of molecular rotors. Proc Natl Acad Sci USA 110: 9225–9230. 10.1073/pnas.1301479110 23690599 PMC3677502

[mbt213471-bib-0061] Howlin, R.P. , Cathie, K. , Hall‐Stoodley, L. , Cornelius, V. , Duignan, C. , Allan, R.N. , *et al* (2017) Low‐dose nitric oxide as targeted anti‐biofilm adjunctive therapy to treat chronic *Pseudomonas aeruginosa* infection in cystic fibrosis. Mol Ther 25: 2104–2116. 10.1016/J.YMTHE.2017.06.021. 28750737 PMC5589160

[mbt213471-bib-0062] Hu, J. , Zhang, N. , Li, L. , Zhang, N. , Ma, Y. , Zhao, C. , *et al* (2018) The synergistic bactericidal effect of vancomycin on UTMD treated biofilm involves damage to bacterial cells and enhancement of metabolic activities. Sci Rep 8: 192 10.1038/s41598-017-18496-3. 29317687 PMC5760522

[mbt213471-bib-0063] Jakobsen, T.H. , Alhede, M. , Hultqvist, L.D. , Bjarnsholt, T. and Givskov, M. (2018) Qualitative and quantitative determination of quorum sensing inhibition in vitro. Meth Mol Biol (Clifton, N.J.) 692, 275–285. 10.1007/978-1-4939-7309-5_21 29130180

[mbt213471-bib-0064] Jamal, M. , Tasneem, U. , Hussain, T. and Andleeb, S. (2015) Bacterial biofilm: its composition, formation and role in human infections. Res Rev 4.

[mbt213471-bib-0065] Jamal, M. , Ahmad, W. , Andleeb, S. , Jalil, F. , Imran, M. , Nawaz, M.A. , *et al* (2018) Bacterial biofilm and associated infections. J Chin Med Assoc 81: 7–11. 10.1016/J.JCMA.2017.07.012. 29042186

[mbt213471-bib-0066] Jang, H. , Rusconi, R. , and Stocker, R. (2017) Biofilm disruption by an air bubble reveals heterogeneous age‐dependent detachment patterns dictated by initial extracellular matrix distribution. NPJ Biofilms Microbiomes 3: 6 10.1038/s41522-017-0014-5. 28649407 PMC5460265

[mbt213471-bib-0067] Jiang, W. , Wang, Y. , Tang, J. , Peng, J. , Wang, Y. , Guo, Q. , *et al* (2016) Low‐intensity pulsed ultrasound treatment improved the rate of autograft peripheral nerve regeneration in rat. Sci Rep 6: 22773 10.1038/srep22773. 27102358 PMC4840319

[mbt213471-bib-0068] Kasimanickam, R.K. , Ranjan, A. , Asokan, G.V. , Kasimanickam, V.R. , and Kastelic, J.P. (2013) Prevention and treatment of biofilms by hybrid‐ and nanotechnologies. Int J Nanomed 8: 2809–2819. 10.2147/IJN.S44100. PMC3739460 23946652

[mbt213471-bib-0069] Khmel, I.A. (2006) Quorum‐sensing regulation of gene expression: fundamental and applied aspects and the role in bacterial communication. Microbiology 75: 390–397. 10.1134/S0026261706040047. 17025169

[mbt213471-bib-0070] Kim, D. , Namen Ii, W. , Moore, J. , Buchanan, M. , Hayes, V. , Myntti, M.F. and Hakaim, A. (2018) Clinical assessment of a biofilm‐disrupting agent for the management of chronic wounds compared with standard of care: a therapeutic approach. Wounds 30, 120–130. 29521641

[mbt213471-bib-0071] Kooiman, K. , Vos, H.J. , Versluis, M. , and de Jong, N. (2014) Acoustic behavior of microbubbles and implications for drug delivery. Adv Drug Deliv Rev 72: 28–48. 10.1016/j.addr.2014.03.003. 24667643

[mbt213471-bib-0072] Kuliasha, C.A. , Finlay, J.A. , Franco, S.C. , Clare, A.S. , Stafslien, S.J. , and Brennan, A.B. (2017) Marine anti‐biofouling efficacy of amphiphilic poly(coacrylate) grafted PDMSe: effect of graft molecular weight. Biofouling 33: 252–267. 10.1080/08927014.2017.1288807. 28270054

[mbt213471-bib-0073] Kumar, A. , Alam, A. , Rani, M. , Ehtesham, N.Z. , and Hasnain, S.E. (2017) Biofilms: Survival and defense strategy for pathogens. Int J Med Microbiol 307: 481–489. 10.1016/J.IJMM.2017.09.016. 28950999

[mbt213471-bib-0074] Laganenka, L. and Sourjik, V. (2017) Autoinducer 2‐dependent Escherichia coli biofilm formation is enhanced in a dual‐species co‐culture. Appl Environ Microbiol 84: AEM.02638‐17 10.1128/aem.02638-17 PMC5812939 29269492

[mbt213471-bib-0075] LaSarre, B. , and Federle, M.J. (2013) Exploiting quorum sensing to confuse bacterial pathogens. Microb Mol Biol Rev 77: 73–111. 10.1128/MMBR.00046-12. PMC3591984 23471618

[mbt213471-bib-0076] Lebeaux, D. , Ghigo, J.‐M. , and Beloin, C. (2014) Biofilm‐related infections: bridging the gap between clinical management and fundamental aspects of recalcitrance toward antibiotics. Microb Mol Biol Rev 78: 510–543. 10.1128/MMBR.00013-14. PMC4187679 25184564

[mbt213471-bib-0077] Lee, J.Y. , Carugo, D. , Crake, C. , Owen, J. , de Saint Victor, M. , Seth, A. , *et al* (2015a) Nanoparticle‐loaded protein‐polymer nanodroplets for improved stability and conversion efficiency in ultrasound imaging and drug delivery. Adv Mat 27: 5484–5492. 10.1002/adma.201502022. 26265592

[mbt213471-bib-0078] Lee, M. , Lee, E.Y. , Lee, D. , and Park, B.J. (2015b) Stabilization and fabrication of microbubbles: applications for medical purposes and functional materials. Soft Matter 11: 2067–2079. 10.1039/C5SM00113G. 25698443

[mbt213471-bib-0079] Lentacker, I. , De Smedt, S.C. , and Sanders, N.N. (2009) Drug loaded microbubble design for ultrasound triggered delivery. Soft Matter 5: 2161 10.1039/b823051j.

[mbt213471-bib-0080] Lentacker, I. , Geers, B. , Demeester, J. , De Smedt, S.C. , and Sanders, N.N. (2010) Design and evaluation of doxorubicin‐containing microbubbles for ultrasound‐triggered doxorubicin delivery: cytotoxicity and mechanisms involved. Mol Ther 18: 101–108. 10.1038/MT.2009.160. 19623162 PMC2839231

[mbt213471-bib-0081] Lentacker, I. , De Cock, I. , Deckers, R. , De Smedt, S.C. , and Moonen, C.T.W. (2014) Understanding ultrasound induced sonoporation: definitions and underlying mechanisms. Adv Drug Deliv Rev 72: 49–64. 10.1016/j.addr.2013.11.008. 24270006

[mbt213471-bib-0082] Liang, X. , Xu, Y. , Gao, C. , Zhou, Y. , Zhang, N. , and Dai, Z. (2018) Ultrasound contrast agent microbubbles with ultrahigh loading capacity of camptothecin and floxuridine for enhancing tumor accumulation and combined chemotherapeutic efficacy. NPG Asia Mat 10: 761–774. 10.1038/s41427-018-0066-x.

[mbt213471-bib-0083] Limoli, D.H. , Jones, C.J. , and Wozniak, D.J. (2015) Bacterial extracellular polysaccharides in biofilm formation and function. Microbiol Spectr 3 10.1128/microbiolspec.MB-0011-2014. PMC4657554 26185074

[mbt213471-bib-0084] Liu, W.‐L. , Jiang, Y.‐L. , Wang, Y.‐Q. , Li, Y.‐X. , and Liu, Y.‐X. (2017) Combined debridement in chronic wounds: A literature review. Chin Nurs Res 4: 5–8. 10.1016/J.CNRE.2017.03.003.

[mbt213471-bib-0085] Lohse, M.B. , Gulati, M. , Johnson, A.D. , and Nobile, C.J. (2017) Development and regulation of single‐ and multi‐species Candida albicans biofilms. Nat Rev Microbiol 16: 19–31. 10.1038/nrmicro.2017.107. 29062072 PMC5726514

[mbt213471-bib-0086] Luan, Y. , Lajoinie, G. , Gelderblom, E. , Skachkov, I. , van der Steen, A.F.W. , Vos, H.J. , *et al* (2014) Lipid shedding from single oscillating microbubbles. Ultrasound Med Biol 40: 1834–1846. 10.1016/J.ULTRASMEDBIO.2014.02.031. 24798388

[mbt213471-bib-0087] Maleki, S. , Almaas, E. , Zotchev, S. , Valla, S. , and Ertesvåg, H. (2016) Alginate biosynthesis factories in pseudomonas fluorescens: localization and correlation with alginate production level. Appl Environ Microbiol 82: 1227–1236. 10.1128/AEM.03114-15. 26655760 PMC4751860

[mbt213471-bib-0088] Malone, M. , Bjarnsholt, T. , McBain, A.J. , James, G.A. , Stoodley, P. , Leaper, D. , *et al* (2017) The prevalence of biofilms in chronic wounds: a systematic review and meta‐analysis of published data. J Wound Care 26: 20–25. 10.12968/jowc.2017.26.1.20. 28103163

[mbt213471-bib-0089] Marsh, P.D. and Zaura, E. (2017) Dental biofilm: ecological interactions in health and disease. J Clin Periodontol 44, S12–S22 10.1111/jcpe.12679 28266111

[mbt213471-bib-0090] McEwan, C. , Owen, J. , Stride, E. , Fowley, C. , Nesbitt, H. , Cochrane, D. , *et al* (2015) Oxygen carrying microbubbles for enhanced sonodynamic therapy of hypoxic tumours. J Control Release 203: 51–56. 10.1016/J.JCONREL.2015.02.004. 25660073

[mbt213471-bib-0091] Michailidis, L. , Bergin, S.M. , Haines, T.P. , and Williams, C.M. (2018) Healing rates in diabetes‐related foot ulcers using low frequency ultrasonic debridement versus non‐surgical sharps debridement: a randomised controlled trial. BMC Res Notes 11: 732 10.1186/s13104-018-3841-4. 30326972 PMC6192336

[mbt213471-bib-0092] Miller, M.B. , and Bassler, B.L. (2001) Quorum sensing in bacteria. Annu Rev Microbiol 55: 165–199. 10.1146/annurev.micro.55.1.165. 11544353

[mbt213471-bib-0093] Mohammad, S. , Mortazavi, J. , Darvish, L. , Abounajmi, M. , Zarei, S. , Zare, T. , *et al* (2015) Alteration of bacterial antibiotic sensitivity after short‐term exposure to diagnostic ultrasound. Iran Red Crescent Med J 17: e26622. 10.5812/ircmj.26622. PMC4698328 26732124

[mbt213471-bib-0094] Mulvana, H. , Stride, E. , Hajnal, J.V. , and Eckersley, R.J. (2010) Temperature dependent behavior of ultrasound contrast agents. Ultrasound Med Biol 36: 925–934. 10.1016/J.ULTRASMEDBIO.2010.03.003. 20447756

[mbt213471-bib-0095] Murphy, C.A. , Houghton, P. , Brandys, T. , Rose, G. , and Bryant, D. (2018) The effect of 22.5 kHz low‐frequency contact ultrasound debridement (LFCUD) on lower extremity wound healing for a vascular surgery population: a randomised controlled trial. Int Wound J 15: 460–472. 10.1111/iwj.12887. 29334176 PMC7949649

[mbt213471-bib-0096] Nesbitt, H. , Sheng, Y. , Kamila, S. , Logan, K. , Thomas, K. , Callan, B. , *et al* (2018) Gemcitabine loaded microbubbles for targeted chemo‐sonodynamic therapy of pancreatic cancer. J Control Release 279: 8–16. 10.1016/J.JCONREL.2018.04.018. 29653222

[mbt213471-bib-0097] Owen, J. , Crake, C. , Lee, J.Y. , Carugo, D. , Beguin, E. , Khrapitchev, A.A. , *et al* (2018) A versatile method for the preparation of particle‐loaded microbubbles for multimodality imaging and targeted drug delivery. Drug Deliv Transl Res 8: 342–356. 10.1007/s13346-017-0366-7. 28299722 PMC5830459

[mbt213471-bib-0098] Paolino, D. , Accolla, M.L. , Cilurzo, F. , Cristiano, M.C. , Cosco, D. , Castelli, F. , *et al* (2017) Interaction between PEG lipid and DSPE/DSPC phospholipids: An insight of PEGylation degree and kinetics of de‐PEGylation. Colloids Surf B Biointerfaces 155: 266–275. 10.1016/J.COLSURFB.2017.04.018. 28460301

[mbt213471-bib-0099] Parsek, M.R. , and Singh, P.K. (2003) Bacterial biofilms: an emerging link to disease pathogenesis. Annu Rev Microbiol 57: 677–701. 10.1146/annurev.micro.57.030502.090720. 14527295

[mbt213471-bib-0100] Persat, A. , Inclan, Y.F. , Engel, J.N. , Stone, H.A. , and Gitai, Z. (2015) Type IV pili mechanochemically regulate virulence factors in *Pseudomonas aeruginosa* . Proc Natl Acad Sci USA 112: 7563–7568. 10.1073/pnas.1502025112. 26041805 PMC4475988

[mbt213471-bib-0101] Pitt, W.G. , and Ross, S.A. (2003) Ultrasound increases the rate of bacterial cell growth. Biotechnol Prog 19: 1038–1044. 10.1021/bp0340685. 12790676 PMC1361254

[mbt213471-bib-0102] Pitt, W.G. , McBride, M.O. , Lunceford, J.K. , Roper, R.J. , and Sagers, R.D. (1994) Ultrasonic enhancement of antibiotic action on gram‐negative bacteria. Antimicrob Agents Chemother 38: 2577–2582. 7872751 10.1128/aac.38.11.2577 PMC188245

[mbt213471-bib-0103] Pitt, W.G. , Husseini, G.A. , and Staples, B.J. (2004) Ultrasonic drug delivery–a general review. Expert Opin Drug Deliv 1: 37–56. 10.1517/17425247.1.1.37. 16296719 PMC1361256

[mbt213471-bib-0104] Povey, M.J.W. , Malcolm, J.W. and Mason, T.J. (1998) Ultrasound in food processing. Blackie Academic & Professional. URL https://books.google.co.uk/books?hl=en%26lr=%26xml:id=eyCB2vJQA9cC%26oi=fnd%26pg=PA151%26dq=principles+of+cavitation+t+g+leighton%26ots=RL_NRYgALv%26sig=px30U89i-W_JI39bsC2rxkn8BMs#v=onepage%26q=principlesofcavitationtgleighton%26f=false.

[mbt213471-bib-0105] Robson, M.C. (2004) Pathophysiology of Chronic Wounds In Surgery in Wounds. Téot L., Banwell P.E., and Ziegler U.E. (eds). Berlin, Heidelberg: Springer, pp. 29–40. 10.1007/978-3-642-59307-9_2

[mbt213471-bib-0106] Rediske, A.M. , Roeder, B.L. , Brown, M.K. , Nelson, J.L. , Robison, R.L. , Draper, D.O. , *et al* (1999) Ultrasonic enhancement of antibiotic action on Escherichia coli biofilms: an in vivo model. Antimicrob Agents Chemother 43: 1211–1214. 10223938 10.1128/aac.43.5.1211 PMC89135

[mbt213471-bib-0107] Reinders, A. , Hee, C.‐S. , Ozaki, S. , Mazur, A. , Boehm, A. , Schirmer, T. , and Jenal, U. (2016) Expression and genetic activation of cyclic DI‐GMP‐specific phosphodiesterases in *Escherichia coli* . J Bacteriol 198: 448–462. 10.1128/JB.00604-15. 26553851 PMC4719445

[mbt213471-bib-0108] Rhoads, D.D. , Wolcott, R.D. , and Percival, S.L. (2008) Biofilms in wounds: management strategies. J Wound Care 17: 502–508. 10.12968/jowc.2008.17.11.31479. 18978690

[mbt213471-bib-0109] Rmaile, A. , Carugo, D. , Capretto, L. , Aspiras, M. , De Jager, M. , Ward, M. , and Stoodley, P. (2014) Removal of interproximal dental biofilms by high‐velocity water microdrops. J Dental Res 93: 68–73. 10.1177/0022034513510945. PMC3872856 24170371

[mbt213471-bib-0110] Runyan, C.M. , Carmen, J.C. , Beckstead, B.L. , Nelson, J.L. , Robison, R.A. , and Pitt, W.G. (2006) Low‐frequency ultrasound increases outer membrane permeability of *Pseudomonas aeruginosa* . J Gen Appl Microbiol 52: 295–301. 10.2323/jgam.52.295. 17310073

[mbt213471-bib-0111] Rutherford, S.T. , and Bassler, B.L. (2012) Bacterial quorum sensing: its role in virulence and possibilities for its control. Cold Spring Harb Perspect Med 2: a012427. 10.1101/cshperspect.a012427. PMC3543102 23125205

[mbt213471-bib-0112] Rychak, J.J. , and Klibanov, A.L. (2014) Nucleic acid delivery with microbubbles and ultrasound. Adv Drug Deliv Rev 72: 82–93. 10.1016/j.addr.2014.01.009. 24486388 PMC4204336

[mbt213471-bib-0113] Saidkhani, V. , Asadizaker, M. , Khodayar, M.J. , and Latifi, S.M. (2016) The effect of nitric oxide releasing cream on healing pressure ulcers. Iran J Nurs Midwifery Res 21: 322–330. 10.4103/1735-9066.180389. 27186212 PMC4857669

[mbt213471-bib-0114] Salta, M. , Capretto, L. , Carugo, D. , Wharton, J.A. , and Stokes, K.R. (2013) Life under flow: a novel microfluidic device for the assessment of anti‐biofilm technologies. Biomicrofluidics American Institute of Physics 7: 64118. 10.1063/1.4850796 PMC3888455 24454610

[mbt213471-bib-0115] Schulz, G. , and Stechmiller, J. (2006) Wound healing and nitric oxide production: too little or too much may impair healing and cause chronic wounds. Int J Low Extrem Wounds 5: 6–8. 10.1177/1534734606286633. 16543205

[mbt213471-bib-0116] Schurr, M.J. (2013) Which bacterial biofilm exopolysaccharide is preferred, Psl or alginate?. J Bacteriol 195: 1623–1626. 10.1128/JB.00173-13. 23417492 PMC3624548

[mbt213471-bib-0117] Shaik, S.S. , Soltau, T.D. , Chaturvedi, G. , Totapally, B. , Hagood, J.S. , Andrews, W.W. , *et al* (2009) Low intensity shear stress increases endothelial ELR+ CXC chemokine production via a focal adhesion kinase‐p38{beta} MAPK‐NF‐{kappa}B pathway. J Biol Chem 284: 5945–5955. 10.1074/jbc.M807205200. 19117939 PMC2645838

[mbt213471-bib-0118] Sharma, P.K. , Gibcus, M.J. , van der Mei, H.C. , and Busscher, H.J. (2005) Influence of fluid shear and microbubbles on bacterial detachment from a surface. Appl Environ Microbiol 71: 3668–3673. 10.1128/AEM.71.7.3668-3673.2005. 16000775 PMC1169060

[mbt213471-bib-0119] Sheikh, S. , Rainger, G.E. , Gale, Z. , Rahman, M. , and Nash, G.B. (2003) Exposure to fluid shear stress modulates the ability of endothelial cells to recruit neutrophils in response to tumor necrosis factor‐α: a basis for local variations in vascular sensitivity to inflammation. Blood 97: 1854–1860. 10.1182/blood.v97.6.1854. 12829609

[mbt213471-bib-0120] Singh, S. , Singh, S.K. , Chowdhury, I. , and Singh, R. (2017) Understanding the mechanism of bacterial biofilms resistance to antimicrobial agents. Open Microbiol J 11: 53–62. 10.2174/1874285801711010053. 28553416 PMC5427689

[mbt213471-bib-0121] Sirsi, S. , and Borden, M. (2009) Microbubble compositions, properties and biomedical applications. Bubble Sci Eng Technol 1: 3–17. 10.1179/175889709X446507. 20574549 PMC2889676

[mbt213471-bib-0122] Stiefel, P. , Schmidt‐Emrich, S. , Maniura‐Weber, K. , and Ren, Q. (2015) Critical aspects of using bacterial cell viability assays with the fluorophores SYTO9 and propidium iodide. BMC Microbiol 15: 36 10.1186/s12866-015-0376-x. 25881030 PMC4337318

[mbt213471-bib-0123] Stride, E. (2008) The influence of surface adsorption on microbubble dynamics. Philos Trans A Math Phys Eng Sci 366: 2103–2115. 10.1098/rsta.2008.0001. 18348975

[mbt213471-bib-0124] Stride, E.P. , and Coussios, C.C. (2009) Cavitation and contrast: the use of bubbles in ultrasound imaging and therapy. Proc Inst Mech Eng H 224: 171–191. 10.1243/09544119JEIM622. 20349814

[mbt213471-bib-0125] Stride, E. , and Edirisinghe, M. (2008) Novel microbubble preparation technologies. Soft Matter 4: 2350 10.1039/b809517p.

[mbt213471-bib-0126] Sun, C. , Sboros, V. , Butler, M.B. , and Moran, C.M. (2014) In vitro acoustic characterization of three phospholipid ultrasound contrast agents from 12 to 43 MHz. Ultrasound Med Biol 40: 541–550. 10.1016/j.ultrasmedbio.2013.10.010. 24361219 PMC3923973

[mbt213471-bib-0127] Swanson, E.J. , Mohan, V. , Kheir, J. , and Borden, M.A. (2010) Phospholipid‐stabilized microbubble foam for injectable oxygen delivery. *Langmuir* Am Chem Soc 26: 15726–15729. 10.1021/la1029432. 20873807

[mbt213471-bib-0128] Taccone, F.S. , Hites, M. , Beumier, M. , Scolletta, S. , and Jacobs, F. (2011) Appropriate antibiotic dosage levels in the treatment of severe sepsis and septic shock. Curr Infect Dis Rep 13: 406–415. 10.1007/s11908-011-0203-y. 21805081

[mbt213471-bib-0129] Taglialegna, A. , Lasa, I. , and Valle, J. (2016) ‘Amyloid Structures as Biofilm Matrix Scaffolds’., Journal of bacteriology. American Society for. Microbiology 198: 2579–2588. 10.1128/JB.00122-16. PMC5019065 27185827

[mbt213471-bib-0130] Taylor, S.F.R. , Brittle, S.A. , Desai, P. , Jacquemin, J. , Hardacre, C. , and Zimmerman, W.A. (2017) Factors affecting bubble size in ionic liquids. Phys Chem Chem Phys 19: 14306–14318. 10.1039/C7CP01725A. 28537605

[mbt213471-bib-0131] Thomas, D.D. , Liu, X. , Kantrow, S.P. , and Lancaster, J.R. Jr (2001) The biological lifetime of nitric oxide: Implications for the perivascular dynamics of NO and O_2_ . Proc Natl Acad Sci USA 98: 355–360. 10.1073/pnas.011379598. 11134509 PMC14594

[mbt213471-bib-0132] Tinkov, S. , Bekeredjian, R. , Winter, G. , and Coester, C. (2009) Microbubbles as ultrasound triggered drug carriers. J Pharmaceut Sci 98: 1935–1961. 10.1002/JPS.21571. 18979536

[mbt213471-bib-0133] Tong, J. , Ding, J. , Shen, X. , Chen, L. , Bian, Y. , Ma, G. , *et al* (2013) Mesenchymal stem cell transplantation enhancement in myocardial infarction rat model under ultrasound combined with nitric oxide microbubbles. PLoS ONE 8: e80186 10.1371/journal.pone.0080186. 24244646 PMC3828189

[mbt213471-bib-0134] Tzu‐Yin, W. , Wilson, K.E. , Machtaler, S. , and Willmann, J.K. (2013) Ultrasound and microbubble guided drug delivery: mechanistic understanding and clinical implications. Curr Pharm Biotechnol 14: 743–752. 24372231 10.2174/1389201014666131226114611 PMC4084724

[mbt213471-bib-0135] Unger, E.C. , Matsunaga, T.O. , McCreery, T. , Schumann, P. , Sweitzer, R. , and Quigley, R. (2002) Therapeutic applications of microbubbles. Eur J Radiol 42: 160–168. 10.1016/S0720-048X(01)00455-7. 11976013

[mbt213471-bib-0136] Valenti, P. , Catizone, A. , Frioni, A. , and Berlutti, F. (2015) Chapter 30 - Lactoferrin and cystic fibrosis airway infection In Diet Exercise Cystic Fibrosis. Watson R. R. (ed). London, UK: Elsevier, pp. 259–270. 10.1016/B978-0-12-800051-9.00030-4.

[mbt213471-bib-0137] Valenti, P. , Frioni, A. , Rossi, A. , Ranucci, S. , De Fino, I. , Cutone, A. , *et al* (2017) Aerosolized bovine lactoferrin reduces neutrophils and pro‐inflammatory cytokines in mouse models of *Pseudomonas aeruginosa* lung infections. Biochem Cell Biol 95: 41–47. 10.1139/bcb-2016-0050. 28129511

[mbt213471-bib-0138] Vohra, P. , and Jasuja, K. (2016) Recent update in ultrasound contrast agents. Int J Sci Rep 2: 274 10.18203/issn.2454-2156.IntJSciRep20163401.

[mbt213471-bib-0139] Walsh, J.W. , Hoffstad, O.J. , Sullivan, M.O. , and Margolis, D.J. (2016) Association of diabetic foot ulcer and death in a population‐based cohort from the United Kingdom. Diabet Med 33: 1493–1498. 10.1111/dme.13054. 26666583

[mbt213471-bib-0140] Wang, R. , Starkey, M. , Hazan, R. , and Rahme, L.G. (2012) Honey's ability to counter bacterial infections arises from both bactericidal compounds and QS inhibition. Fron Microbiol 3: 144 10.3389/fmicb.2012.00144. PMC3323871 22514552

[mbt213471-bib-0141] Wang, C. , Yang, F. , Xu, Z. , Shi, D. , Chen, D. , Dai, J. , *et al* (2013) Intravenous release of NO from lipidic microbubbles accelerates deep vein thrombosis resolution in a rat model. Thrombosis Res 131: e31–e38. 10.1016/j.thromres.2012.11.002. 23199547

[mbt213471-bib-0142] Watters, C.M. , Burton, T. , Kirui, D.K. , and Millenbaugh, N.J. (2016) Enzymatic degradation of in vitro *Staphylococcus aureus* biofilms supplemented with human plasma. Infect Drug Resist 9: 71–78. 10.2147/IDR.S103101. 27175088 PMC4854256

[mbt213471-bib-0143] Williams, D. , Enoch, S. , Miller, D. , Harris, K. , Price, P. , and Harding, K.G. (2005) Effect of sharp debridement using curette on recalcitrant nonhealing venous leg ulcers: a concurrently controlled, prospective cohort study. Wound Repair Regen 13: 131–137. 10.1111/j.1067-1927.2005.130203.x. 15828937

[mbt213471-bib-0144] Witte, M.B. , and Barbul, A. (2002) Role of nitric oxide in wound repair. Am J Surg 183: 406–412. 11975928 10.1016/s0002-9610(02)00815-2

[mbt213471-bib-0145] Wolcott, R.D. , Rhoads, D.D. , and Dowd, S.E. (2008) Biofilms and chronic wound inflammation. J Wound Care 17: 333–341. 10.12968/jowc.2008.17.8.30796. 18754194

[mbt213471-bib-0146] Wolcott, R.D. , Kennedy, J.P. , and Dowd, S.E. (2009) Regular debridement is the main tool for maintaining a healthy wound bed in most chronic wounds. J Wound Care 18: 54–56. 10.12968/jowc.2009.18.2.38743. 19418781

[mbt213471-bib-0147] Wu, J. , and Nyborg, W.L. (2008) Ultrasound, cavitation bubbles and their interaction with cells. Adv Drug Deliv Rev 60: 1103–1116. 10.1016/J.ADDR.2008.03.009. 18468716

[mbt213471-bib-0148] Wu, H. , Moser, C. , Wang, H.‐Z. , Høiby, N. , and Song, Z.‐J. (2015) Strategies for combating bacterial biofilm infections. Int J Oral Sci 7: 1–7. 10.1038/ijos.2014.65. 25504208 PMC4817533

[mbt213471-bib-0149] Xu, L.‐C.C. , Wo, Y. , Meyerhoff, M.E. , and Siedlecki, C.A. (2017) Inhibition of bacterial adhesion and biofilm formation by dual functional textured and nitric oxide releasing surfaces. Acta Biomater 51: 53–65. 10.1016/j.actbio.2017.01.030. 28087484 PMC5346060

[mbt213471-bib-0150] Yazdanpanah, L. , Nasiri, M. and Adarvishi, S. (2015) Literature review on the management of diabetic foot ulcer WJD 5 th Anniversary Special Issues (4): diabetes‐related complications. World J Diabet, 6, 37–53. 10.4239/wjd.v6.i1.37 PMC4317316 25685277

[mbt213471-bib-0151] Ye, J. , He, H. , Gong, J. , Dong, W. , Huang, Y. , Wang, J. , *et al* (2013) Ultrasound‐mediated targeted microbubbles: a new vehicle for cancer therapy. Front Chem Sci Eng 7: 20–28. 10.1007/s11705-013-1311-z.

[mbt213471-bib-0152] Yusof, N.S.M. , Babgi, B. , Alghamdi, Y. , Aksu, M. , Madhavan, J. , and Ashokkumar, M. (2016) Physical and chemical effects of acoustic cavitation in selected ultrasonic cleaning applications. Ultrason Sonochem 29: 568–576. 10.1016/J.ULTSONCH.2015.06.013. 26142078

[mbt213471-bib-0153] Zhu, H.‐X. , Cai, X.‐Z. , Shi, Z.‐L. , Hu, B. , and Yan, S.‐G. (2014) Microbubble‐mediated ultrasound enhances the lethal effect of gentamicin on planktonic *Escherichia coli* . Biomed Res Int 2014: 142168 10.1155/2014/142168. 24977141 PMC4052079

[mbt213471-bib-0154] Zhuang, X. , Dávila‐Contreras, E.M. , Beaven, A.H. , Im, W. , and Klauda, J.B. (2016) ‘An extensive simulation study of lipid bilayer properties with different head groups, acyl chain lengths, and chain saturations. Biochimica et Biophysica Acta (BBA) – Biomembranes 1858: 3093–3104. 10.1016/J.BBAMEM.2016.09.016. 27664502

